# Risk nomogram for papillary thyroid microcarcinoma with central lymph node metastasis and postoperative thyroid function follow-up

**DOI:** 10.3389/fendo.2024.1395900

**Published:** 2024-10-28

**Authors:** Yuting Huang, Pengwei Lou, Hui Li, Yinhui Li, Li Ma, Kai Wang

**Affiliations:** ^1^ Department of Medical Administration, Traditional Chinese Medicine Hospital Affiliated to Xinjiang Medical University, Urumqi, China; ^2^ Department of Big Data, College of Information Engineering, Xinjiang Institute of Engineering, Urumqi, China; ^3^ Department of Endocrine, Traditional Chinese Medicine Hospital Affiliated to Xinjiang Medical University, Urumqi, China; ^4^ College of Public Health, Xinjiang Medical University, Urumqi, China

**Keywords:** papillary thyroid microcarcinoma, central lymph node metastasis, risk factors, nomogram, thyroid function

## Abstract

**Background:**

The treatment for papillary thyroid microcarcinoma (PTMC) is controversial. Central lymph node metastasis (CLNM) is one of the main predictors of recurrence and survival, accurate preoperative identification of CLNM is essential for surgical protocol establishment for PTMC. The objective of this study was to establish a nomogram to predict the possibility of CLNM in PTMC patients.

**Methods:**

A total of 3023 PTMC patients were randomly divided into two groups by a ratio of 7 to 3, the training group (n = 2116) and validation group (n = 907). The LASSO regression model and multivariate logistic regression analysis were performed to examine risk factors associated with CLNM. A nomogram for predicting CLNM was established and internally validated. Meanwhile, we follow-up the serum thyroid function FT3, FT4, TSH, Tg, TGAb and TPOAb in 789 PTMC patients for 4 years after surgery and compared the differences between the CLNM (+) and CLNM (-) groups, respectively.

**Results:**

The LASSO regression model and multivariate logistic regression analysis showed that younger age, lower BMI, being male, location in the lower pole, calcification, 1 ≥ diameter ≥ 0.5 cm, multifocality lesions, extra thyroidal extension (ETE), enlargement of central lymph node (ECLN), lateral lymph node metastasis (LLNM) and higher carcinoembryonic antigen were the ultimate risk factors for determining CLNM. A nomogram for predicting CLNM was constructed based on the influencing factors and internally validated. By establishing the prediction model, the AUC of CLNM in the training and validation groups were 0.73 (95% CI, 0.70-0.76) and 0.75 (95% CI, 0.71-0.79) respectively. Results of the DCA showed that the model is clinically useful when deciding on intervention in the most range of the threshold probability. A 4-year follow-up of thyroid function showed that FT3 and FT4 remained at stable levels after 3 months postoperative and were higher in the CLNM (+) group than in the CLNM (-) group. Hypothyroidism appeared predominantly within 3 months after surgery. The overall incidence of the CLNM (+) group and CLNM (-) groups were 16.46% and 12.04%, respectively.

**Conclusion:**

The nomogram model constructed in this study has a good predictive effect on CLNM in PTMC patients and provides a reasonable reference for clinical treatment.

## Introduction

Papillary thyroid microcarcinoma (PTMC) is defined as malignant thyroid tumors of ≤ 1cm in diameter, which accounts for approximately 30-60% of the papillary thyroid carcinoma (PTC) (1–3). In recent years, the incidence of thyroid microcarcinoma has increased rapidly, mainly attributed to the application of routine high-resolution ultrasonography (USG) and other imaging techniques (4). In the 5th edition of the WHO classification criteria for thyroid tumors, the subtype classification based on tumor size has been eliminated, mainly due to the fact that factors such as pathological subtypes, aggressiveness, and lymph node metastasis of thyroid cancer have a greater impact on the prognosis, and physicians develop individualized treatment plans accordingly (5).

Most PTMCs are known to be inert and have a favorable prognosis, with a long-term survival rate of exceeding 99% (6). However, some PTMCs still have high-risk features at the time of diagnosis, for example, cervical lymph node metastasis and extrathyroidal extension (ETE) are closely associated with an increased risk of distant metastasis, high local recurrence, and death (3, 7). Central lymph node metastasis (CLNM) was found in 40-60% of PTMC patients in most series by using standard pathology technique (8, 9). The 2015 American Thyroid Association (ATA) guidelines for differentiated thyroid cancer stated that central lymph node dissection (CLND) should be considered when the preoperative ultrasound shows a significant metastatic lymph node (cN1) or if they are palpable at the time of examination. Routine prophylactic CLND is not recommended for non-invasive, T1 or T2 and clinically node-negative (cN0) PTC (10). The need for routine prophylactic CLND in patients without clinical evidence of CLNM, especially for those with unilateral lesions, remains controversial (11).

The Chinese and Japanese PTC guidelines recommend that prophylactic CLND should adequately protect the parathyroid gland and the recurrent laryngeal nerve (RLN) (12, 13). Considering the prediction of CLNM is critical in potentially deciding on the surgical approach. Therefore, it is crucial to accurately assess the status of the cervical lymph nodes. Although preoperative high-resolution ultrasound (HUS) plays an important role in CLNM evaluation, it has quite low sensitivity (14). Therefore, it is of great significance to develop a new method for accurately evaluating the status of the cervical lymph nodes, in order to provide guidance for a personalized surgical approach. Many studies have reported on the preoperative clinicopathologic risk factors of CLNM for PTMC, but the results are inconsistent (15–17).

Some studies have only predicted risk factors for CLNM in patients with PTMC, but long-term follow-up studies of thyroid function after surgery are scarce (18, 19). Currently, there are no comprehensive and systematic reports on the prediction of preoperative CLNM risk, postoperative trends in thyroid function indicators and complication rates in patients with PTMC, especially comparing the differences between CLNM (+) (represents the occurrence of CLNM) and CLNM (–) (indicates that no CLNM occurred) groups have hardly reported, which is a limitation for PTMC patients to understand their postoperative progression.

The aim of our study was to establish a practical nomogram for preoperative prediction of the likelihood of CLNM based on clinical, hematological, USG and pathological features in order to determine the surgical extent and therapeutic strategy for patients with PTMC. We also analyzed the trends of postoperative thyroid function indicators, the incidence of postoperative complications of subclinical hypothyroidism and hypothyroidism, and compared the differences between the CLNM (+) and CLNM (–) groups, which provided a reference for clinicians to develop postoperative treatment plans.

## Materials and methods

### Study design and participants

Clinical case data of patients who underwent thyroid surgery and were diagnosed with PTMC postoperatively from January 2015 to December 2022 in Traditional Chinese Medicine Hospital of Xinjiang Medical University were retrospectively selected. The variables included patient age, sex, BMI, maximum tumor size, history at diagnosis, thyroid function, USG parameters, pathological results and other indicators. The exclusion criteria were as follows: (1) patients who underwent secondary or further thyroid surgery; (2) patients who did not undergo radical surgery or CLND; (3) previously diagnosed other concomitant malignancies, such as breast cancer, lung cancer, colon cancer, and other cancers; (4) patients who also had other types of thyroid carcinoma, such as medullary, follicular and anaplastic thyroid carcinomas; (5) clinically and/or pathologically detected distant metastasis, such as secondary malignant tumor of bone, liver, and other body parts; (6) patients lost to follow-up or with incomplete clinical information.

### Information collection

The possible risk factors of PTMC with CLNM analyzed in this study can be divided into 4 categories including 40 variables.

The general clinical conditions include: gender, age, ethnicity, marital status, body mass index (BMI), diabetes, heart disease, smoking, drinking, family history of cancer, family history of thyroid disease. All of these factors can be obtained from hospitalized records.

Biochemical variables recorded include thyroid function [free triiodothyronine (FT3), free thyroxine (FT4), thyroid stimulating hormone (TSH), thyroglobulin (Tg)], thyroid antibody [thyrotropin receptor antibody (TRAb), Anti-thyroglobulin antibodies (TgAb), thyroid peroxidase antibodies (TPOAb)], blood lipid [triglyceride (TG), cholesterol (CHOL)], tumor markers [alpha fetoprotein (AFP), carcinoembryonic antigen (CEA), calcitonin (Ct)]. The results of these biochemical indicators are exported from the hospital information system.

A GE-E11 ultrasound system (GE Medical System, USA) with a linear array probe was used to acquire ultrasound images in the frequency range of 9-11MHz. All patients were in the supine position with the lower shoulder pillow neck extended to better expose the inferior thyroid rim. Both thyroid lobes and isthmus of the thyroid were scanned in the transverse and longitudinal planes. Longitudinal and transverse images of the thyroid were obtained according to American College of Radiology accreditation standards. The thyroid nodule ultrasound chart was prejudged by two experienced sonographers.

The following USG parameters of the nodules were recorded as follows: (1) margin, (2) shape, (3) echogenicity, (4) calcification, (5) diffuse, (6) color doppler flow imaging (CDFI), (7) tumor position: location_Upper/Middle/Lower, location_Left/Right/Isthmus, (8) ECLN.

The clinicopathological test results included: (1) diameter, (2) hashimoto thyroditis, (3) nodular goiter, (4) ETE, (5) multifocality, (6) bilateral, (7) lateral lymph node metastasis (LLNM). Postoperative histopathologic evaluations were performed by pathologists experienced in thyroid pathology.

This judgement criteria of the study were as follows: (1) malignant tumors were considered to be multifocal when more than one malignant tumor foci were found in the same or different lobes of the gland; (2) if the same patient had multiple lesions, the malignant tumor with the largest diameter was selected as the research object. When the diameter was the same, the higher Thyroid Imaging Reporting and Data System (TI-RADS) level would be selected. The maximum tumor diameter was categorized in two groups: the small group (< 0.5 cm) and the large group (0.5-1.0 cm); (3) ETE was confirmed when there was evidence of a malignant tumor invading adipose tissue, banded muscle, larynx, trachea, esophagus, recurrent laryngeal nerve, prevertebral fascia or blood vessels; (4) lateral lymph node dissection (LLND), including levels II-V, was performed only in cases with clinically evident lateral neck lymph node metastasis. Assessed by the experienced physician, the patients without LLND were classified as non-occurrence LLNM; (5) the range of TGAb and TPOAb actual detection results is wide, being 0-4000 IU/ml and 0-600 IU/ml respectively. In this study, these were set as dichotomous variables: TGAb was divided into Normal (≤ 115 IU/ml) and Abnormal (> 115 IU/ml) groups; TPOAb was also divided into Normal (≤ 34 IU/ml) and Abnormal (> 34 IU/ml) groups.

### Follow-up of thyroid function indicators

In order to clarify the trend of thyroid function in PTMC patients at different postoperative periods and to compare the differences between the CLNM (+) and the CLNM (-) groups, thyroid function indicators were retrospectively collected in this study. FT3, FT4, TSH, Tg, TgAb, and TPOAb in PTMC patients during the following consecutive periods: preoperatively, within 3 days, 1 month, 3 months, 6 months, 1 year, 2 years, 3 years, and 4 years postoperatively. By the end of 2022, those eligible for follow-up 4 years thyroid function indicator data were PTMC patients who underwent surgery during 2015-2018. Data were obtained from thyroid function markers at the time the patients underwent regular follow-up of patients during outpatient or hospitalization. Patients with omissions or missing data are excluded, and those with high compliance, completeness, and continuity of thyroid function indicators were retained for analysis. In accordance with guidelines for the diagnosis and management of subclinical thyroid disorders and the standard practices in hospital laboratories, the normal ranges of thyroid function indicators were determined as follows: 3.1 ≤ FT3 ≤ 6.8 pmol/L, 12 ≤ FT4 ≤ 22 pmol/L, 0.27 ≤ TSH ≤ 4.2 uIU/ml, 3.5 ≤ Tg ≤ 77 ng/ml, 0 ≤ TGAb ≤ 115 IU/ml, 0 ≤ TPOAb ≤ 34 IU/ml. Subclinical hypothyroidism is defined as an elevation in serum TSH level above the upper limit of the reference range and a normal FT4 level. Overt hypothyroidism is defined as an increase in serum TSH level and a decrease in FT4 level.

### Data screening

**Figure 1:**
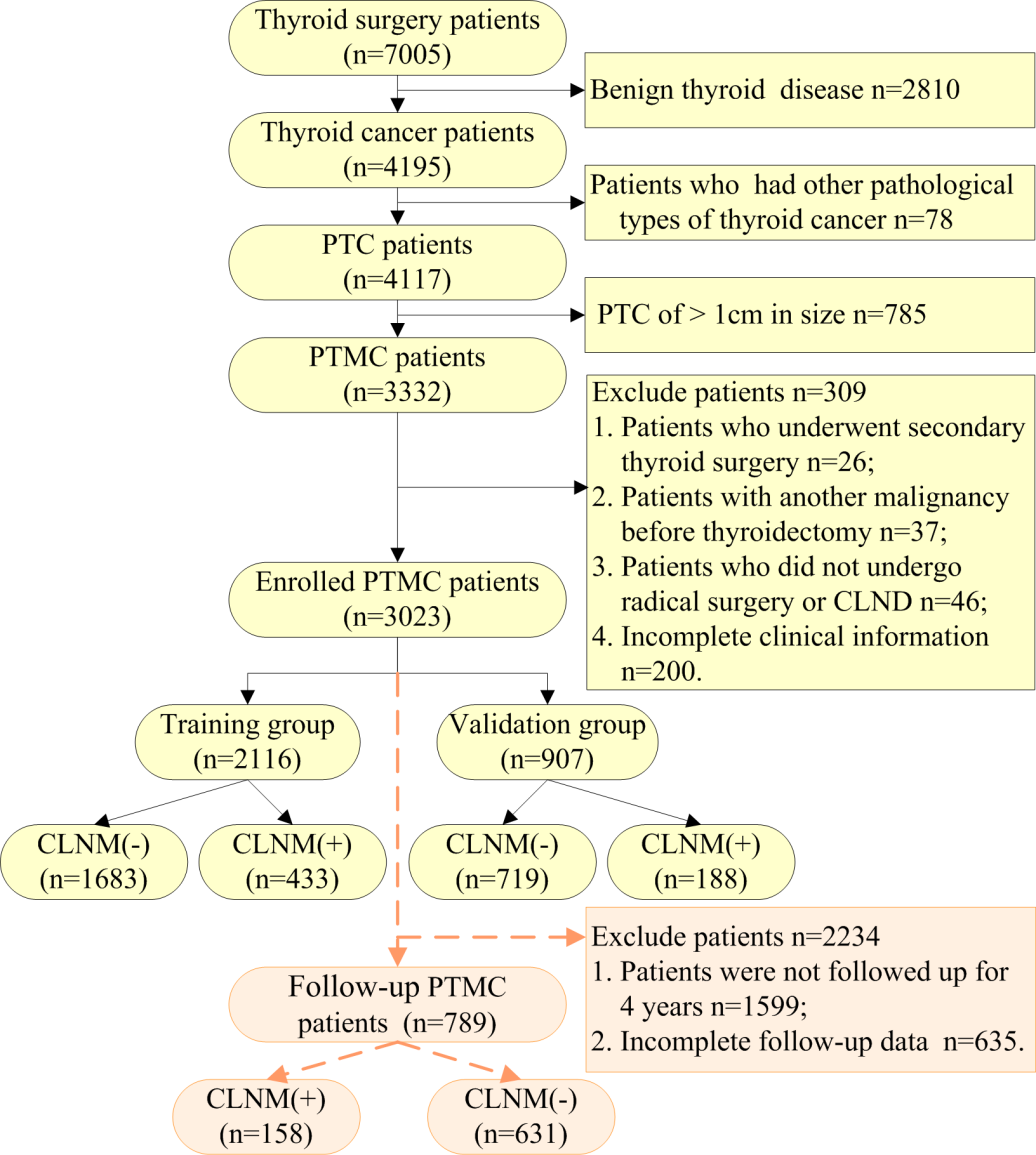
Flow diagram of the data screening.

Between January 2015 and December 2022, there were 7005 thyroid surgery in-patients based on the medical record data of Traditional Chinese Medicine Hospital Affiliated to Xinjiang Medical University. According to strict exclusion criteria, 3023 PTMC cases were included in this study in the end, 621 had CLNM, as shown in **Figure 1**. To better verify the predictive power of the model and to analyze the possibility of CLNM in PTMC, 3023 cases were randomly divided into two groups by a ratio of 7 to 3, 2116 were in the training group and 907 in the validation group.

A total of 789 patients with complete serum thyroid function indicators at 4-year postoperative follow-up among the patients with PTMC, including 158 with CLNM (+) and 631 with CLNM (-), as shown in **Figure 1**. There were 1424 eligible patients for PTMC surgery from 2015-2018, with a complete follow-up rate of 55.41%.

The study was approved by the Medical Ethics Committee of Traditional Chinese Medicine Hospital Affiliated to Xinjiang Medical University (IRB No. 2022XE0171). All participants gave written informed consent for their clinical records to be used in this study.

### Thyroid surgery and pathologic analysis

During surgery, the tumors were sent for frozen sections and confirmed as PTMC. After rediagnosis, these patients underwent thyroidectomy and cervical lymph node dissection, and the lymph nodes were sent for frozen sections to confirm CLNM and LLNM. After surgery, the remaining specimens were sent for paraffin sections for final confirmation of PTMC, CLNM and LLNM. Two or more experienced pathologists microscopically reviewed and cross-checked all pathology specimens. Principles of thyroidectomy: total thyroidectomy was performed in patients with extraglandular invasion, bilateral or multiple lesions, and bilateral cervical lymph node metastases, and ipsilateral and isthmus resection were performed in patients with unilateral intrathyroidal tumors. Principles of cervical lymph node dissection: central lymph nodes (level VI) were routinely dissection, and lateral compartment lymph nodes (levels II-V) with evidence of lymph node metastasis should be dissection. Upper mediastinal lymph nodes (level VII) should be also cleared if there are enlarged lymph nodes based on neck imaging. The recurrent laryngeal nerves should be directly visualized throughout the node clearance process to avoid injury. The parathyroid gland and their blood supply should be retained.

A total of 3023 patients with PTMC who underwent surgical treatment were included in this study, of which 1360 patients underwent total thyroidectomy, 1341 patients underwent unilateral lobectomy + isthmus resection, 314 patients underwent unilateral lobectomy + isthmus + partial resection of contralateral lobectomy, and 8 patients underwent bilateral partial lobectomy + isthmus resection. Lymph node dissection was performed in zone VI only in 2798 patients (ipsilateral 1164 patients and bilateral 1154 patients) and in zones II, III, IV and VI in 224 patients (ipsilateral 5 patients and bilateral 219 patients).

### Statistical analysis

The open source R software, Version 4.3.3 (https://www.r-project.org) and the Statistical Package for the Social Sciences (SPSS) for Windows, Version 21.0 (SPSS Inc., Chicago, IL, United States) were used for data analysis and modeling in this study. When contrasting the baseline information and clinical features between the modeling group and the control group, the chi-square test or Fisher’s exact test were used for categorical variables, expressed by actual cases and percentages (%); the student’s t-test or Wilcoxon rank sum test was used for continuous variables, shown by mean ± standard deviation (SD). After assessing risk factors by the least absolute shrinkage and selection operator (LASSO) regression model, the non-zero variate was considered significant. A multivariate logistic regression model for prediction was then established, and the coefficient, odds ratio (OR) with 95% confidence interval (CI) and *p*-values of variables were established at the same time; the statistical significance variables (*p*-values< 0.05) were the influence factors. All statistical tests were two sided, and *p*-values< 0.05 were considered statistically significant.

A nomogram, based on influencing factors determined by the multivariate logistic regression model, was established by rms/pROC pack in R software, which was used to evaluate the risk of CLNM preoperatively and gave every variable to a score between 0 and 100. The goodness of fit test of the model between modeling and validation groups was assessed by receiver operating characteristic (ROC) curve and calibration curve. The ROC curve was plotted to quantify and contrast the risk of PTMC with CLNM between forecasting and observing cases. The reasonable range of area under ROC curve (AUC) is 0.5 to 1, the closer the value is to 1, the stronger the model prediction ability. The calibration curve, as a very important criteria, was used to assess how well the model fits the real data. Our conclusion from the contained 1000 bootstrap samples: the smaller the mean absolute error, the higher the accuracy in forecasting was. Vice versa, the lower accuracy showed the model may over- or underestimate the risk of illness. A Hosmer-Lemeshow goodness-of-fit test was also performed to assess predictive ability; the larger the p-value, the better the predictive ability. The DCA was drawn to make the clinical decision benefit in predicting the risk of PTMC with CLNM.

## Results

### Baseline characteristics

In our study, a total of 3023 PTMC patients were recruited according to the inclusion and exclusion criteria; the average age was 47.33 ± 10.39 years, there were 2381 (78.76%) females and 642 (21.24%) males (ratio 3.71:1). CLNM and non-CLNM were observed in 621 (20.54%) and 2402 (79.46%) cases respectively.

A total of 2116 patients with PTMC were included in the training group consisting of 1665 (78.69%) females and 451 (21.31%) males (ratio 3.69:1), and the average age was 47.27 ± 10.44 years. CLNM and non-CLNM were observed in 433 (20.46%) and 1683 (79.54%) cases respectively. Meanwhile, 907 PTMC patients were assigned to the validation group, there were 716 (78.94%) females and 191 (21.06%) males (ratio 3.75:1), and the average age was 47.46 ± 10.27 years. CLNM and non-CLNM were observed in 188 (20.73%) and 719 (79.27%) cases respectively.

We collected 43 indexes to identify the risk factors for CLNM in PTMC. There were no statistically significant differences (*P* > 0.05) in general clinical data, biochemical indicators, ultrasonic characteristics and clinicopathological results between the training and the validation group, except for the Ct, as shown in **Table 1**.

**Table 1:** Baseline clinical characteristics in patients with PTMC.

Characteristics	Total n = 3023	Training group n = 2116	Validation group n = 907	$x^{2}$ or *t*-test	*P* value
Group				0.027	0.869
CLNM (+)	621 (20.54)	433 (20.46)	188 (20.73)		
CLNM (-)	2402 (79.46)	1683 (79.54)	719 (79.27)		
Gender				0.025	0.875
Male	642 (21.24)	451 (21.31)	191 (21.06)		
Female	2381 (78.76)	1665 (78.69)	716 (78.94)		
Age (years)	47.33 ± 10.39	47.27 ± 10.44	47.46 ± 10.27	0.478	0.633
BMI (kg/m2)	24.91 ± 3.54	24.91 ± 3.55	24.90 ± 3.51	-0.028	0.978
Ethnicity				0.022	0.989
Han	2334 (77.21)	1635 (77.27)	699 (77.07)		
Uygur	313 (10.35)	218 (10.30)	95 (10.47)		
Other nationalities	376 (12.44)	263 (12.43)	113 (12.46)		
Marital status				2.975	0.226
Single	118 (3.90)	91 (4.30)	27 (2.98)		
Married	2768 (91.57)	1929 (91.16)	839 (92.50)		
Divorced/Windowed	137 (4.53)	96 (4.54)	41 (4.52)		
Smoking				0.060	0.807
Yes	317 (10.49)	220 (10.39)	97 (10.69)		
No	2706 (89.51)	1896 (89.61)	810 (89.31)		
Drinking				1.373	0.241
Yes	275 (9.10)	184 (8.70)	91 (10.03)		
No	2748 (90.90)	1932 (91.30)	816 (89.97)		
Hashimoto thyroditis				0.264	0.607
Yes	762 (25.21)	539 (25.47)	223 (24.59)		
No	2261 (74.79)	1577 (74.53)	684 (75.41)		
Nodular goiter				0.740	0.390
Yes	1222 (40.42)	866 (40.93)	356 (39.25)		
No	1801 (59.58)	1250 (59.07)	551 (60.75)		
Hypertension				0.003	0.960
Yes	655 (21.67)	459 (21.69)	196 (21.61)		
No	2368 (78.33)	1657 (78.31)	711 (78.39)		
Diabetes				0.227	0.634
Yes	285 (9.43)	203 (9.59)	82 (9.04)		
No	2738 (90.57)	1913 (90.41)	825 (90.96)		
Heart disease				3.243	0.072
Yes	193 (6.38)	124 (5.86)	69 (7.61)		
No	2830 (93.62)	1992 (94.14)	838 (92.39)		
Family history of cancer				0.140	0.708
Yes	468 (15.48)	331 (15.64)	137 (15.10)		
No	2555 (84.52)	1785 (84.36)	770 (84.90)		
Family history of thyroid disease				0.016	0.899
Yes	75 (2.48)	52 (2.46)	23 (2.54)		
No	2948 (97.52)	2064 (97.54)	884 (97.46)		
Diameter (cm)	0.52 ± 0.25	0.52 ± 0.25	0.51 ± 0.25	-0.784	0.433
< 0.5	1259 (41.65)	878 (41.49)	381 (42.01)	0.069	0.793
0.5-1	1764 (58.35)	1238 (58.51)	526 (57.99)		
ETE				0.347	0.556
Yes	281 (9.30)	201 (9.50)	80 (8.82)		
No	2742 (90.70)	1915 (90.50)	827 (91.18)		
LLNM				0.231	0.631
Yes	159 (5.26)	114 (5.39)	45 (4.96)		
No	2864 (94.74)	2002 (94.61)	862 (95.04)		
ECLN				3.818	0.051
Yes	357 (11.81)	234 (11.06)	123 (13.56)		
No	2666 (88.19)	1882 (88.94)	784 (86.44)		
Bilateral				0.535	0.465
Yes	513 (16.97)	366 (17.30)	147 (16.21)		
No	2510 (83.03)	1750 (82.70)	760 (83.79)		
Multifocality				0.665	0.415
Yes	763 (25.24)	543 (25.66)	220 (24.26)		
No	2260 (74.76)	1573 (74.34)	687 (75.74)		
Location_UML				0.122	0.941
Upper pole	652 (21.57)	457 (21.60)	195 (21.50)		
Middle third	1226 (40.56)	854 (40.36)	372 (41.01)		
Lower pole	1145 (37.87)	805 (38.04)	340 (37.49)		
Location_LRI				2.172	0.337
Left lobe	1385 (45.82)	952 (45.00)	433 (47.74)		
Right lobe	1525 (50.45)	1086 (51.32)	439 (48.40)		
Isthmus	113 (3.73)	78 (3.68)	35 (3.86)		
Diffuse				0.447	0.504
Yes	511 (16.90)	364 (17.20)	147 (16.21)		
No	2512 (83.10)	1752 (82.80)	760 (83.79)		
Echogenicity				0.080	0.778
Hypoechoic	3008 (99.50)	2106 (99.53)	902 (99.45)		
Isoechoic/Hyperechoic	15 (0.50)	10 (0.47)	5 (0.55)		
Margin				0.837	0.360
Clear	852 (28.18)	586 (27.69)	266 (29.33)		
Unclear	2171 (71.82)	1530 (72.31)	641 (70.67)		
Shape				2.763	0.096
Regular	463 (15.32)	309 (14.60)	154 (16.98)		
Irregular	2560 (84.68)	1807 (85.40)	753 (83.02)		
CDFI				0.239	0.625
Yes	1626 (53.79)	1132 (53.50)	494 (54.47)		
No	1397 (46.21)	984 (46.50)	413 (45.53)		
Calcification				0.113	0.737
Yes	1083 (35.83)	754 (35.63)	329 (36.27)		
No	1940 (64.17)	1362 (64.37)	578 (63.73)		
TG (mmol/L)	1.49 ± 1.01	1.50 ± 1.04	1.46 ± 0.94	-1.163	0.245
CHOL (mmol/L)	4.41 ± 0.92	4.41 ± 0.93	4.40 ± 0.90	-0.097	0.923
FT3 (pmol/L)	4.63 ± 1.01	4.63 ± 1.05	4.63 ± 0.92	-0.167	0.868
FT4 (pmol/L)	15.84 ± 3.08	15.82 ± 3.17	15.88 ± 2.84	0.451	0.652
TSH (uIU/ml)	2.67 ± 2.17	2.66 ± 2.14	2.69 ± 2.23	0.435	0.664
Tg (ng/ml)	21.34 ± 51.34	21.45 ± 50.81	21.06 ± 52.56	-0.192	0.847
TRAb (IU/L)	0.69 ± 1.30	0.68 ± 1.24	0.70 ± 1.42	0.309	0.757
TgAb (IU/ml)	105.83 ± 334.89	103.75 ± 323.43	110.65 ± 360.36	0.518	0.604
Normal [≤ 115]	2506 (82.90)	1748 (82.61)	758 (83.57)	0.416	0.519
Abnormal [> 115]	517 (17.10)	368 (17.39)	149 (16.43)		
TPOAb (IU/ml)	44.43 ± 98.23	42.88 ± 94.91	48.01 ± 105.54	1.260	0.208
Normal [≤ 34]	2509 (83.00)	1758 (83.08)	751 (82.80)	0.035	0.851
Abnormal [> 34]	514 (17.00)	358 (16.92)	156 (17.20)		
AFP (ng/ml)	3.36 ± 2.76	3.33 ± 2.23	3.42 ± 3.73	0.803	0.508
CEA (ng/ml)	1.56 ± 1.06	1.54 ± 1.01	1.58 ± 1.17	0.839	0.402
Ct (pg/ml)	1.19 ± 1.92	1.23 ± 2.15	1.10 ± 1.21	-2.112	0.035

### Characteristics selection

**Figure 2:**
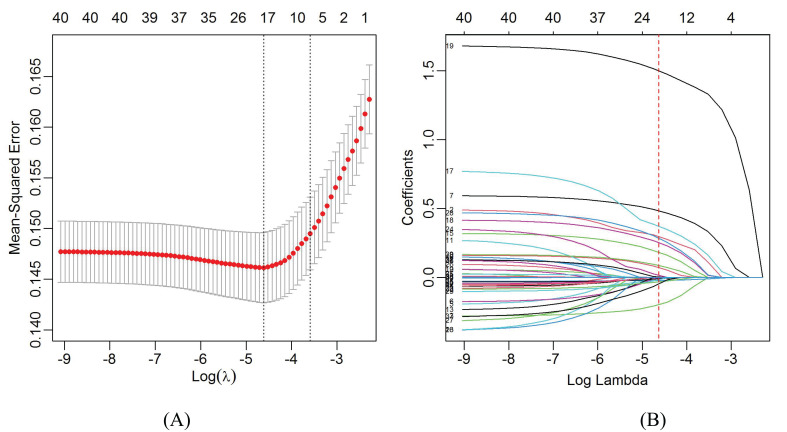
Demographic and clinical feature selection using the least absolute shrinkage and selection operator (LASSO) binary logistic regression model. **(A)** Optimal parameters (λ) selection in the LASSO model used tenfold cross-validation via minimum criteria. The Mean-Squared Error was plotted as a function of log(λ). The dotted red curve indicates the average Mean-Squared Error values. The area under the receiver operation characteristic curve was plotted versus log(λ). Dotted vertical lines were plotted at the optimal values by using the minimum criteria and the 1 standard error of the minimum criteria. **(B)** LASSO coefficient profiles of the 20 features. A coefficient profile plot was produced against the log(λ) sequence. A vertical line was plotted at the value selected using tenfold cross validation, where optimal lambda resulted in 20 features with non-zero coefficients.

For the study, the LASSO regression was used for feature selection in the training group. With the change of the penalty coefficient λ, the variables included in the model were gradually reduced. The λ in the LASSO model was selected via tenfold-validation based on minimum criteria (lambda.min = 0.0099). At the same time, 18 non-zero coefficient characteristics (**Figures 2A, B**) were selected to build the multivariate logistic regression analysis, including age, gender, BMI, marital status, hashimoto thyroditis, location_UML, calcification, diameter, multifocality, ETE, ECLN, LLNM, TG, FT3, TSH, Tg, TPOAb, CEA.

**Figure 3:**
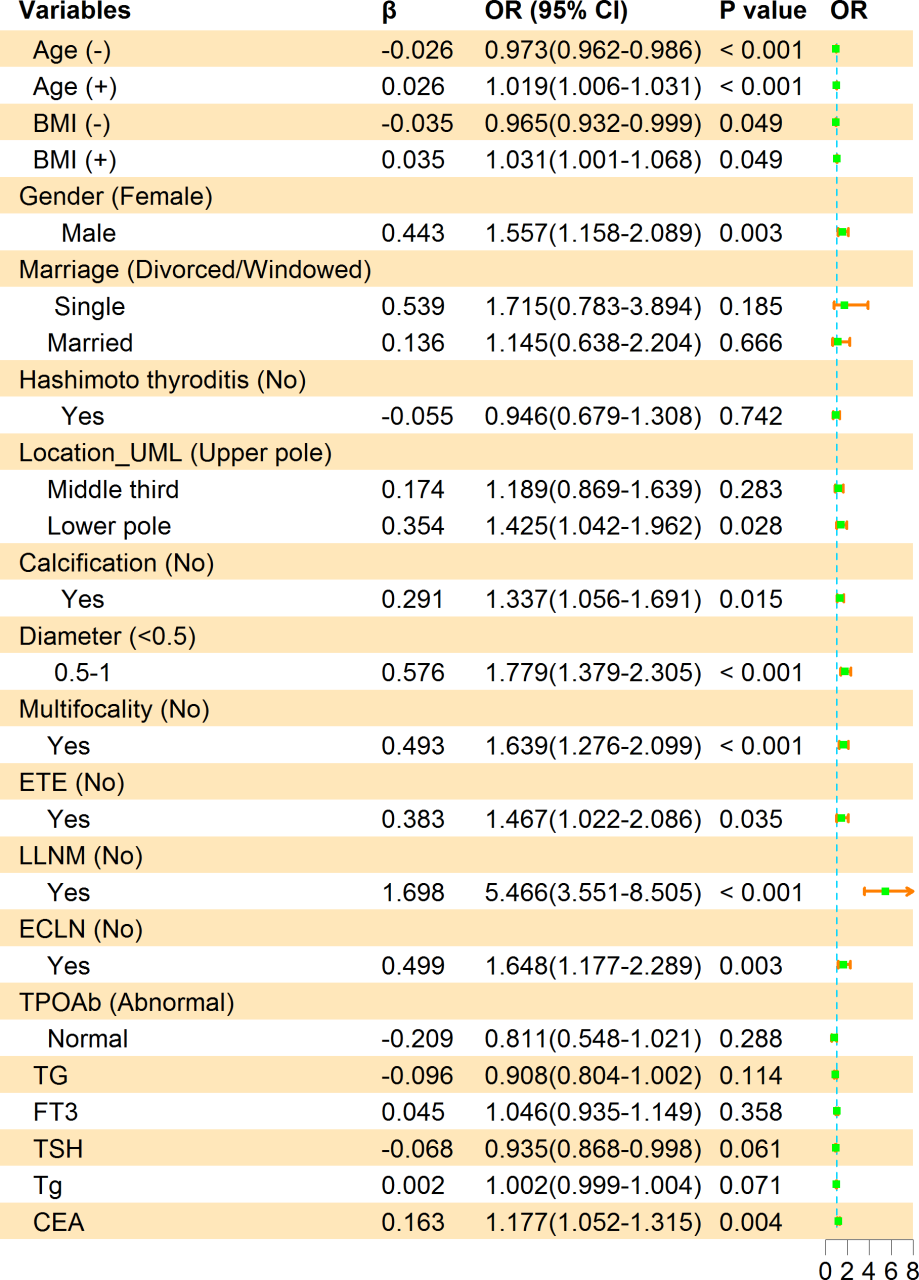
Multivariate logistic regression analysis of factors associated with CLNM in the training group. β is the regression coefficient; CI, confidence interval; OR, odds ratio. The symbols (+) indicate positive correlation and (-) represent negative correlation.

The multivariate logistic regression analysis concluded that younger age (OR = 1.02, 95% CI, 1.01-1.03, *P<* 0.001), Lower BMI (OR = 1.03, 95% CI, 1.00-1.07, *P* = 0.049), being male (OR = 1.56, 95% CI, 1.16-2.09, *P* = 0.003), location in lower pole (OR = 1.43, 95% CI, 1.04-1.96, *P* = 0.028), calcification (OR = 1.34, 95% CI, 1.06-1.69, *P* = 0.015), 1 ≥ diameter ≥ 0.5 cm (OR = 1.78, 95% CI, 1.38-2.31, *P<* 0.001), multifocality lesions (OR = 1.64, 95% CI, 1.28-2.10, *P<* 0.001), ETE (OR = 1.47, 95% CI, 1.02-2.09, *P =* 0.035), ECLN (OR = 1.65, 95% CI, 1.18-2.29, *P =* 0.003), LLNM (OR = 5.47, 95% CI, 3.55-8.51, *P<* 0.001) and CEA (OR = 1.18, 95% CI, 1.05-1.32, *P* = 0.003) were the independent risk factors of PTMC patients with CLNM. It should be emphasized that age (-) and BMI (-) were correlated with CLNM negatively, the higher the value, the lower the risk of CLNM. We calculated the risk value of Age (+) and BMI (+) by conversion of the dependent variables. The detailed results are shown in **Figure 3**. Meanwhile, we list the distribution characteristics of the 11 risk factors in CLNM (+) group and CLNM (-) group respectively in **Table 2**. The differences were statistically significant (*P<* 0.05).

**Table 2:** Risk factors characteristics associated with CLNM in PTMC.

Variables	Training group n (%)			Validation group n (%)			Total n (%)		
	CLNM (+)	CLNM (-)	*P* value	CLNM (+)	CLNM (-)	*P* value	CLNM (+)	CLNM (-)	*P* value
Total	433 (100)	1683 (100)		188 (100)	719 (100)		621 (100)	2402 (100)	
Gender			< 0.001			0.004			< 0.001
Male	130 (30.02)	321 (19.07)		54 (28.72)	137 (19.05)		184 (29.63)	458 (19.07)	
Female	303 (69.98)	1362 (80.93)		134 (71.28)	582 (80.95)		437 (70.37)	1944 (80.93)	
Location_UML			0.029			0.033			0.001
Upper pole	79 (18.24)	378 (22.46)		30 (15.96)	165 (22.95)		109 (17.55)	543 (22.61)	
Middle third	167 (38.57)	687 (40.82)		74 (39.36)	298 (41.45)		241 (38.81)	985 (41.01)	
Lower pole	187 (43.19)	618(36.72)		84 (44.68)	256 (35.60)		271 (43.64)	874 (36.38)	
Calcification			< 0.001			0.044			< 0.001
No	245 (56.58)	1117 (66.37)		108 (57.45)	470 (65.37)		353 (56.84)	1587 (66.07)	
Yes	188 (43.42)	566 (33.63)		80 (42.55)	249 (34.63)		268 (43.16)	815 (33.93)	
Diameter			< 0.001			< 0.001			< 0.001
<0.5	112 (25.87)	766 (45.51)		30 (15.96)	351 (48.82)		142 (22.87)	1117 (46.50)	
0.5-1	321 (74.13)	917 (54.49)		158 (84.04)	368 (51.18)		479 (77.13)	1285 (53.50)	
Multifocality			< 0.001			< 0.001			< 0.001
No	273 (63.05)	1300 (77.24)		116 (61.70)	571 (79.42)		389 (62.64)	1871 (77.89)	
Yes	160 (36.95)	383 (22.76)		72 (38.30)	148 (20.58)		232 (37.36)	531 (22.11)	
ETE			< 0.001			< 0.001			< 0.001
No	361 (83.37)	1554 (92.33)		154 (81.91)	673 (93.60)		515 (82.93)	2227 (92.71)	
Yes	72 (16.63)	129 (7.67)		34(18.08)	46 (6.40)		106 (17.07)	175 (7.29)	
LLNM			< 0.001			< 0.001			< 0.001
No	360 (83.14)	1642 (97.56)		154 (81.91)	708 (98.47)		514 (82.77)	2350 (97.84)	
Yes	73 (16.86)	41 (2.44)		34 (18.09)	11 (1.53)		107 (17.23)	52 (2.16)	
ECLN			<0.001			0.006			<0.001
No	352 (81.29)	1530 (90.91)		151 (80.32)	633 (88.04)		503 (81.00)	2163 (90.05)	
Yes	81 (18.71)	153 (9.09)		37 (19.68)	86 (11.96)		118 (19.00)	239 (9.95)	
Age	44.44 ± 11.44	47.99 ± 10.04	<0.001	44.87 ± 10.42	48.14 ± 10.13	<0.001	44.57 ± 11.13	48.04 ± 10.06	<0.001
BMI	24.55 ± 3.59	24.94 ± 3.54	0.002	24.59 ± 3.66	24.93 ± 3.47	0.027	24.57 ± 3.61	24.94 ± 3.51	0.009
CEA	1.66 ± 1.09	1.51 ± 0.98	0.012	1.65 ± 1.40	1.56 ± 1.09	0.104	1.66 ± 1.19	1.53 ± 1.02	0.017

### Nomogram of CLNM in PTMC patients

**Figure 4:**
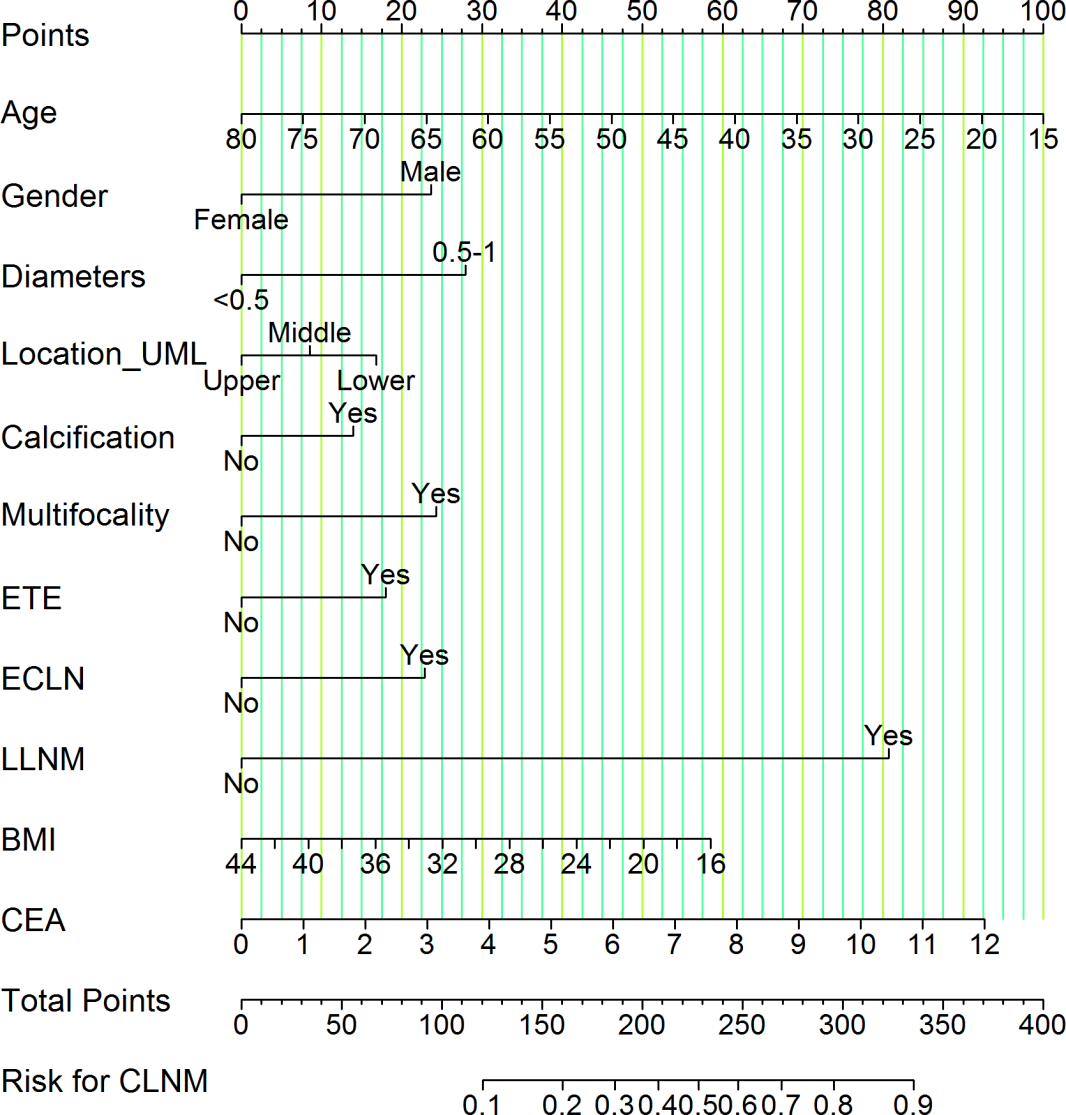
Nomogram of CLNM risk in patients with PTMC. We calculated the corresponding points according to the assigning principle. Gender: 23.75 points for male; Diameters: 28.00 points for 0.5-1 cm; Location_UML: 17.00 points for lower pole and 8.75 points for middle third; Calcification: 13.75 points for yes; Multifocality: 24.00 points for yes; ETE: 18.00 points for yes; ECLN: 22.50 points for yes; LLNM: 81.00 points for yes; Age: point range is 0-100, with an increase of 1.54 points for each additional year of age. BMI: Based on a scale of 0-58.50, 2.09 points are added to the score for every 1 kg/m^2^ reduction in BMI. CEA: on a scale from 0 to 92.50, each 1 ng/mL raise in CEA is associated with an increase of 7.71 points. Each risk factor score add up to the total points, which represents the risk for CLNM.

Based on LASSO and multivariate logistic regression analysis, we produced a nomogram model consisting of 11 factors, which were used to estimate the risk of CLNM in PTMC patients. According to the scored ruler as shown in **Figure 4**, each variety has a corresponding point value on each variable axis: all the points were added up to obtain the overall points. The sum of these numbers is located on the total points axis to express the incidence of the risk for CLNM. The higher the total points, the greater the possibility of CLNM risk. In this study, the risk of PTMC combined with CLNM ranged from 0% to 90%, as assessed by 11 risk factors.

### Validation of nomogram for the CLNM

**Figure 5:**
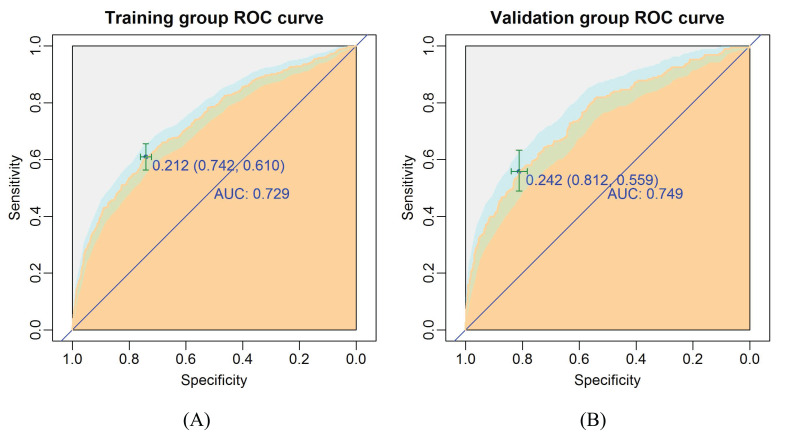
The ROC curves of the nomogram for CLNM risk. **(A)** Training group; **(B)** Validation group. The burly wood color represents the area under the ROC curve; 95% CI of the ROC curve was colored light blue.

The ROC curve was drawn to predict the nomogram effect (**Figures 5A, B**). The area under the ROC curve (AUC) of the training group was 0.73 (95% CI, 0.70-0.76), the diagnostic cut-off point was 0.21 (sensitivity, 0.61, specificity, 0.74). The AUC of the validation group was 0.75 (95% CI, 0.71-0.79), the diagnostic cut-off point was 0.24 (sensitivity, 0.56, specificity, 0.81). In addition, the AUC value of the validation group was only 0.02 higher than that of the training group, which indicates that the model has good prediction discrimination in both groups.

**Figure 6:**
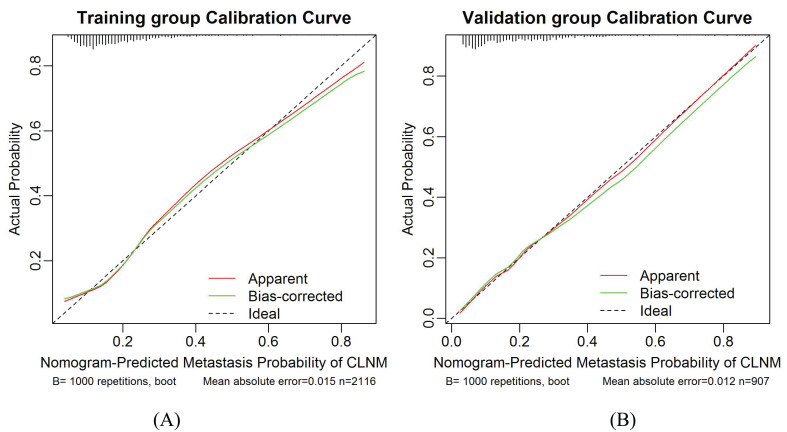
Calibration curves for the training group and validation group models. **(A)** Training group. **(B)** Validation group. The diagonal dotted line represents the ideal prediction by the perfect nomogram. The green solid line represents the performance of the nomogram. The closer the green solid line is to the diagonal dotted line, the stronger the predictive ability of the model. The red solid line indicates the apparent predictive accuracy.

The calibration plots present a good agreement between the actual and predicted metastasis probability with an additional 1000 bootstraps, the mean absolute error of the training and validation groups were 0.015 and 0.012, respectively (**Figures 6A, B**). Meanwhile, the calibration of the prediction model was evaluated by Hosmer-Lemeshow goodness of fit test: when *P* > 0.05, the calibration ability of the model is good. The *P* values of the training and validation groups calibration curves were 0.95 and 0.60, respectively, which indicated that the nomogram model was well consistent between prediction and actual values.

### DCA curve of the nomogram

**Figure 7:**
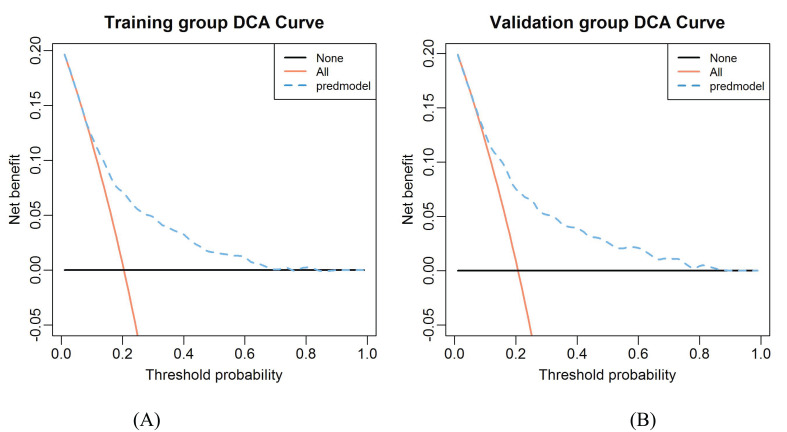
The decision curve analysis for CLNM in the constructed risk nomogram. **(A)** Training group. **(B)** Validation group.

Results of DCA in training and validation groups were shown in **Figures 7A, B**. The DCA showed that using the nomogram model to predict the occurrence of CLNM risk would be better than using another two conditions (all patients and none of the patients treated). This means that the model is clinically useful when intervention is decided on in the range of the threshold probability between 0.09-0.82 and 0.07-0.86 in the training and validation groups respectively.

### Novel risk stratification based on the predictive nomogram

**Figure 8:**
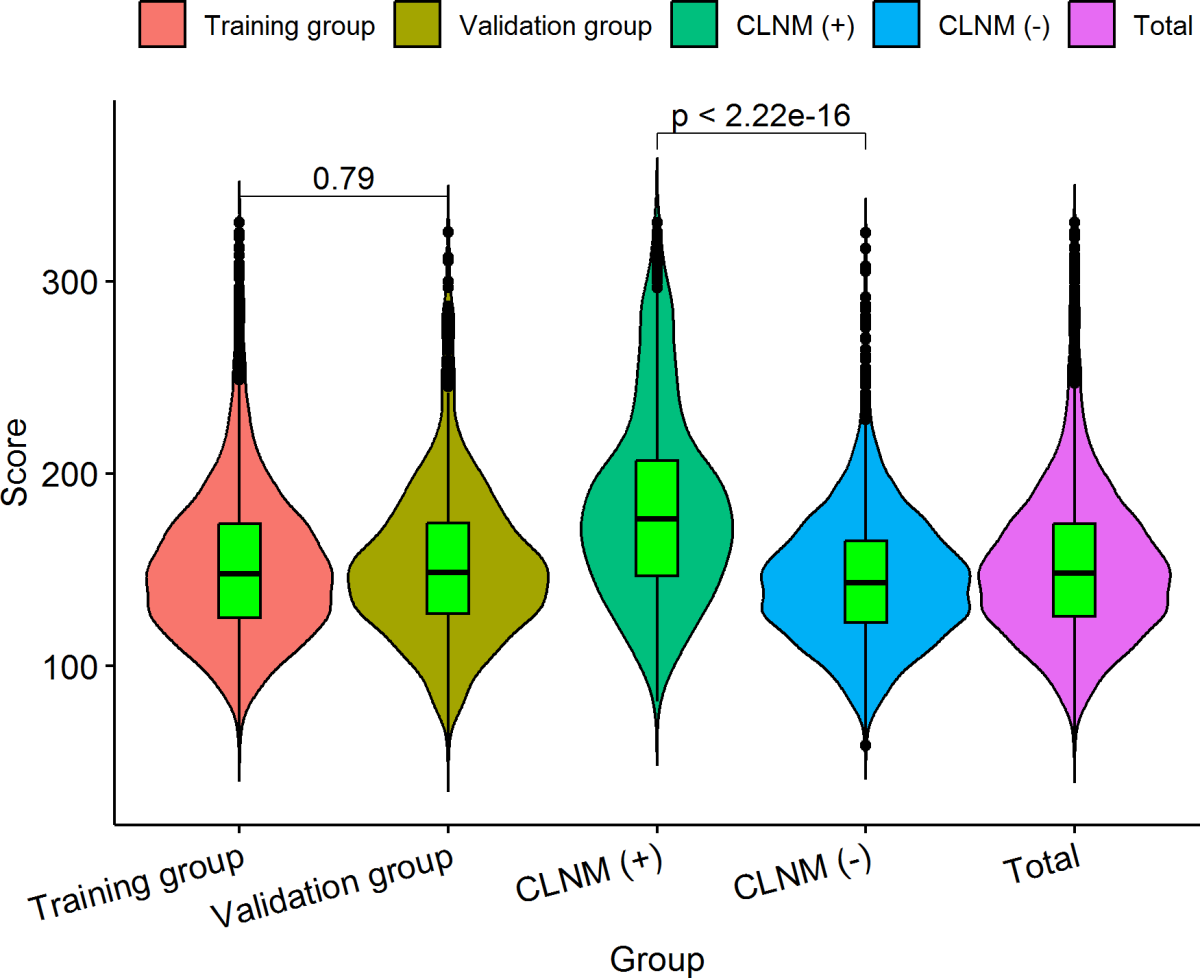
The distribution characteristic of the total CLNM risk points. The length of the violin chart represents the range of points, the width indicates the frequency of points, and the black horizontal line in the boxplot represents the average points. We calculated that the average points of the training group is 153.22 ± 41.11 (61.12-330.68), and 152.92 ± 40.07 (58.80-325.38) for the validation group; there was no significant difference (*P* = 0.790) between the two groups. The average points of the CLNM (+) group 182.73 ± 49.72 (81.60-330.68) was significantly higher than that of CLNM (-) group 145.48 ± 34.22 (58.80-325.19), with a statistically significant difference (*P<* 0.001).

Each variable contained in the nomogram has its corresponding risk point, and the total points calculated for all patients can quantitatively predict their respective CLNM risk. The distribution characteristic of the total CLNM risk points are shown in **Figure 8**, and the risk value is shown in **Figure 4**. We therefore determined three cut-off values (150, 200 and 240) by using recursive partition analysis, and classified three subgroups as follows: (1) extreme low-risk group (total points ≤ 150, risk ≤ 18%), low-risk group (151 ≤ total points ≤ 200, risk 19-36%), (2) moderate-risk group (201 ≤ total points ≤ 240, risk 37-58%), (3) high-risk group (total points > 250, risk > 58%). In the training and validation groups, the rates of CLNM for extreme low-, low-, moderate-, and high-risk groups were 10.57%, 24.64%, 45.89%, 69.88% and 10.65%, 22.92%, 53.57%, 83.33%, respectively, showing a gradual upward trend. We further studied whether the relative risk of CLNM in low-, moderate-, and high-risk groups was significantly different from one another. The Chi-square test showed that there were significant differences between all groups (*P<* 0.05). The results of the training and validation groups confirmed that there were no significant differences in the same category group (*P* > 0.05). As shown in **Table 3**.

**Table 3:** Metastasis risk stratification of patients with PTMC based on risk scores of the nomogram model.

Nomogram	ELR	LR	MR	HR	Total	*P* value	ELR-LR	ELR-MR	ELR-HR	LR-MR	LR-HR	MR-HR
	≤ 150	151-200	201-240	≥ 241			*P* value	*P* value	*P* value	*P* value	*P* value	*P* value
Training group												
CLNM (+)	118 (10.57)	190 (24.64)	67 (45.89)	58 (69.88)	433	< 0.001	< 0.001	< 0.001	< 0.001	< 0.001	< 0.001	< 0.001
CLNM (-)	998 (89.43)	581 (75.36)	79 (54.10)	25 (30.12)	1683							
Total	1116	771	146	83	2116							
Validation group												
CLNM (+)	51 (10.65)	77 (22.92)	30 (53.57)	30 (83.33)	188	< 0.001	< 0.001	< 0.001	< 0.001	< 0.001	< 0.001	0.003
CLNM (-)	428 (89.35)	259 (77.08)	26 (46.43)	6 (16.67)	719							
Total	479	336	56	36	907							
*P* value	0.965	0.537	0.328	0.125								

ELR, extreme low risk; LR, low risk; MR, moderated risk; HR, high risk.

### Follow-up of postoperative serum thyroid function indicators

We retrospectively collected complete follow-up data on serum thyroid function indicators for 4 years after surgery in 789 patients with PTMC, of whom 158 (20.03%) developed CLNM and 631 (79.97%) did not, which was not statistically significant difference when compared with the overall distribution of CLNM occurrence ( $\chi^{2}=0.068$ , *P* = 0.794). The differences in FT3, TSH, and TPOAb in the preoperative CLNM (+) group were statistically significant compared with the CLNM (-) group, which were also previously screened by LASSO regression.

**Figure 9:**
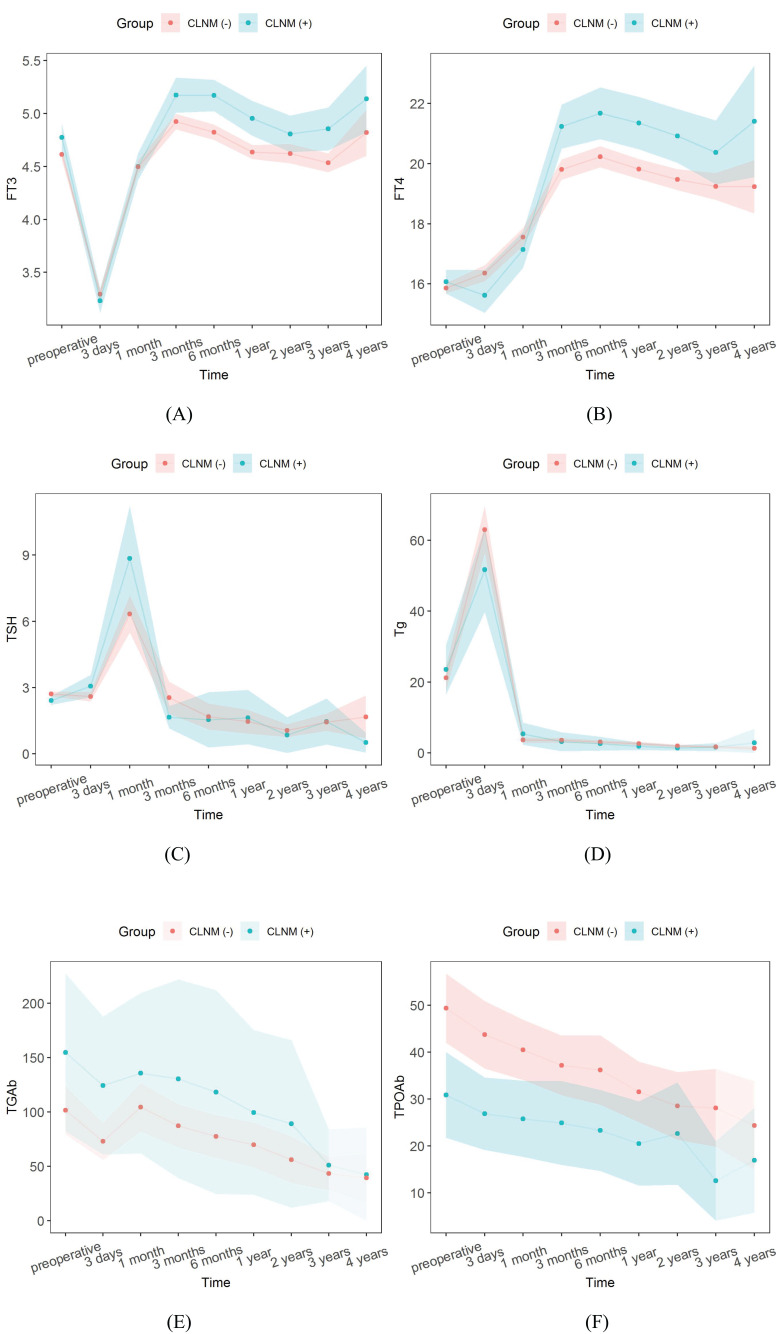
Trends in thyroid function outcomes of PTMC patients in the CLNM (+) and CLNM (-) groups within 4 years follow-up. **(A)** FT3, **(B)** FT4, **(C)** TSH, **(D)** Tg, **(E)** TGAb, and **(F)** TPOAb.

The follow-up data of serum thyroid function indicators in PTMC patients during 4 years after surgery and the results of comparison between CLNM (+) and CLNM (-) groups are shown in **Table 4**, **Figure 9**. FT3 decreased to the lowest value 3.28 ± 0.77 pmol/L at 3 days postoperatively, then gradually increased to a relatively stable state of 4.97 ± 0.97 pmol/L at 3 months postoperatively, and was significantly higher in the CLNM (+) group than in the CLNM (-) group (*P*< 0.05). FT4 remained elevated for 3 months postoperatively and then stabilized at 20.09 ± 4.44 pmol/L, which was higher than the preoperative level of 15.90 ± 2.52 pmol/L, the CLNM (+) group was higher than the CLNM (-) group (*P*< 0.05). The mean TSH level reached the highest value 6.84 ± 13.19 uIU/ml at 1 month postoperatively, then gradually decreased and was maintained at a stable level (2.36 ± 8.52 uIU/ml) at 3 months postoperatively, there was no statistically significant difference between the CLNM (+) and CLNM (-) groups (*P* > 0.05). The mean Tg level reached a maximum value of 60.72 ± 93.59 ng/ml at 3 days postoperatively, then gradually decreased and remained at a stable level 4.00 ± 13.95 ng/ml at 1 month postoperatively, which was lower than the preoperative level of 21.65 ± 50.23 ng/ml, and the difference between the CLNM (+) and CLNM (-) groups was not statistically significant (*P* > 0.05). TGAb showed a gradual decrease, with no statistical difference between the CLNM (+) and CLNM (-) groups (*P* > 0.05). TPOAb also showed a gradual decline, with CLNM (+) group being lower than the CLNM (-) group, with a statistically significant difference within 1 year postoperatively (*P*< 0.05).

**Table 4:** 4-year follow-up results of thyroid function in the CLNM (+) and CLNM (-) groups of PTMC patients.

Variables	Time	Total n = 789	CLNM (+) n = 158	CLNM (-) n = 631	*t*	*P*
FT3						
	Preoperative	4.65 ± 0.76	4.77 ± 0.92	4.61 ± 0.72	-2.613	0.009
	3 days	3.28 ± 0.77	3.23 ± 0.82	3.29 ± 0.76	1.029	0.304
	1 month	4.49 ± 0.91	4.50 ± 0.91	4.49 ± 0.91	-0.060	0.953
	3 months	4.97 ± 0.97	5.17 ± 1.06	4.92 ± 0.94	-2.898	0.004
	6 months	4.89 ± 0.89	5.17 ± 0.88	4.82 ± 0.88	-4.113	<0.001
	1 year	4.70 ± 0.81	4.95 ± 0.92	4.64 ± 0.77	-3.966	<0.001
	2 years	4.66 ± 0.90	4.81 ± 0.84	4.62 ± 0.91	-1.774	0.077
	3 years	4.60 ± 0.76	4.85 ± 0.81	4.53 ± 0.73	-3.073	0.002
	4 years	4.89 ± 0.83	5.14 ± 0.64	4.82 ± 0.87	-2.444	0.015
FT4						
	Preoperative	15.90 ± 2.52	16.07 ± 2.79	15.85 ± 2.45	-0.999	0.319
	3 days	16.21 ± 3.95	15.62 ± 4.20	16.35 ± 3.87	2.319	0.021
	1 month	17.47 ± 4.27	17.14 ± 4.33	17.56 ± 4.25	1.216	0.224
	3 months	20.09 ± 4.44	21.23 ± 4.64	19.80 ± 4.34	-3.634	<0.001
	6 months	20.50 ± 4.46	21.67 ± 5.11	20.21 ± 4.23	-3.098	0.002
	1 year	20.13 ± 4.07	21.35 ± 4.92	19.84 ± 3.78	-3.193	0.002
	2 years	19.75 ± 3.70	20.92 ± 4.33	19.47 ± 3.47	-2.997	0.003
	3 years	19.47 ± 3.78	20.37 ± 4.25	19.24 ± 3.62	-2.153	0.032
	4 years	19.71 ± 3.67	21.41 ± 3.74	19.23 ± 3.53	-2.281	0.025
TSH						
	Preoperative	2.63 ± 1.60	2.41 ± 1.46	2.71 ± 1.63	2.366	0.018
	3 days	2.68 ± 3.20	3.06 ± 3.65	2.59 ± 3.07	-1.676	0.095
	1 month	6.84 ± 13.19	8.84 ± 17.05	6.33 ± 11.98	-1.946	0.053
	3 months	2.36 ± 8.52	1.66 ± 3.21	2.54 ± 9.38	1.947	0.052
	6 months	1.65 ± 7.13	1.54 ± 7.40	1.68 ± 7.07	0.215	0.829
	1 year	1.49 ± 6.96	1.63 ± 9.32	1.45 ± 6.26	-0.200	0.842
	2 years	1.01 ± 2.68	0.84 ± 1.48	1.05 ± 2.90	0.667	0.505
	3 years	1.44 ± 3.42	1.46 ± 4.17	1.43 ± 3.21	-0.072	0.943
	4 years	1.41 ± 3.46	0.50 ± 0.69	1.67 ± 3.87	1.268	0.208
Tg						
	Preoperative	21.65 ± 50.23	23.53 ± 50.24	21.17 ± 50.25	-0.587	0.557
	3 days	60.72 ± 93.59	51.67 ± 85.14	63.02 ± 95.55	1.520	0.129
	1 month	4.00 ± 13.95	5.40 ± 22.09	3.65 ± 10.95	-1.081	0.281
	3 months	3.48 ± 11.16	3.18 ± 17.05	3.55 ± 9.13	0.377	0.706
	6 months	2.97 ± 7.49	2.59 ± 11.39	3.06 ± 6.15	0.659	0.510
	1 year	2.48 ± 5.94	1.89 ± 5.94	2.62 ± 5.93	1.231	0.219
	2 years	1.84 ± 3.83	1.39 ± 3.75	1.95 ± 3.85	1.241	0.215
	3 years	1.68 ± 3.59	1.61 ± 4.55	1.70 ± 3.31	0.185	0.854
	4 years	1.62 ± 4.37	2.81 ± 8.03	1.28 ± 2.54	-0.794	0.437
TGAb						
	Preoperative	112.36 ± 366.07	154.76 ± 516.55	101.57 ± 316.11	-1.381	0.169
	3 days	83.52 ± 287.12	124.43 ± 437.55	72.94 ± 232.27	-1.545	0.124
	1 month	110.75 ± 333.02	135.69 ± 473.19	104.43 ± 287.07	-0.799	0.425
	3 months	95.69 ± 311.07	130.47 ± 522.49	87.36 ± 233.66	-0.912	0.363
	6 months	85.68 ± 282.86	118.39 ± 482.41	77.46 ± 204.41	-0.846	0.399
	1 year	75.82 ± 254.62	99.54 ± 385.92	69.81 ± 209.00	-0.751	0.454
	2 years	62.50 ± 220.99	89.07 ± 325.64	56.19 ± 188.03	-0.819	0.415
	3 years	45.00 ± 110.47	51.17 ± 116.20	43.44 ± 109.23	-0.441	0.659
	4 years	40.01 ± 74.23	42.23 ± 75.33	39.31 ± 74.74	-0.127	0.899
TPOAb						
	Preoperative	45.61 ± 98.53	30.84 ± 65.06	49.37 ± 105.07	3.099	0.002
	3 days	40.27 ± 91.03	26.87 ± 53.40	43.74 ± 98.18	3.139	0.001
	1 month	37.54 ± 77.43	25.78 ± 52.07	40.51 ± 82.39	2.800	0.005
	3 months	34.78 ± 71.25	24.89 ± 51.30	37.16 ± 75.09	2.199	0.029
	6 months	33.62 ± 71.60	23.31 ± 44.61	36.21 ± 76.72	2.234	0.026
	1 year	29.27 ± 62.81	20.50 ± 45.95	31.52 ± 66.29	1.966	0.051
	2 years	27.39 ± 60.48	22.63 ± 46.08	28.53 ± 63.43	0.738	0.461
	3 years	24.99 ± 53.69	12.54 ± 8.85	28.12 ± 59.52	3.541	<0.001
	4 years	22.58 ± 29.67	16.95 ± 20.23	24.37 ± 32.08	0.842	0.403

Thyroid function indicators FT3, FT4, and Tg varied within the normal range, TSH exceeded the maximum normal level at 1 month postoperatively, the CLNM (+) group with TGAb and the CLNM (-) group with TPOAb exceeded the highest normal level within 6 months and then gradually decreased to the normal range.

**Figure 10:**
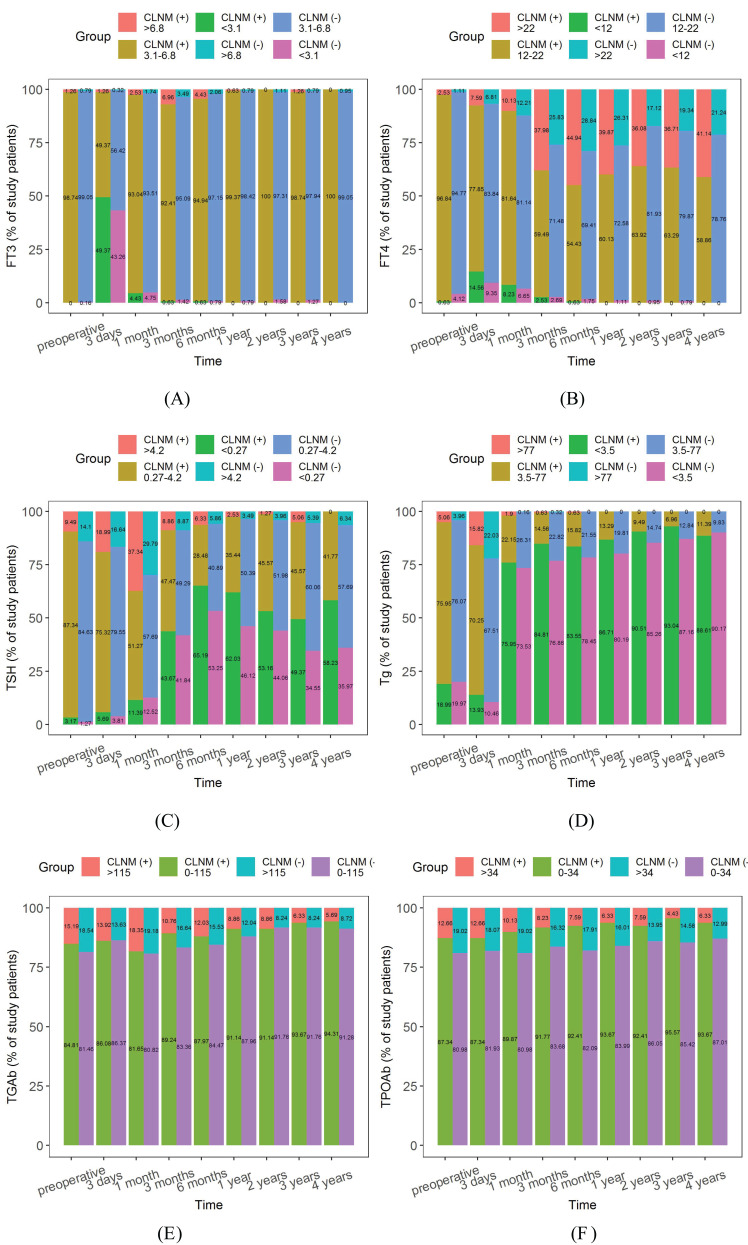
Composition ratio of normal and abnormal reference ranges of postoperative thyroid function indicators in the CLNM (+) and CLNM (-) groups of PTMC patients with 4 years follow-up. **(A)** FT3, **(B)** FT4, **(C)** TSH, **(D)** Tg, **(E)** TGAb, and **(F)** TPOAb.

The composition ratio of normal and abnormal reference ranges of serum thyroid function indicators during the 4 years postoperative follow-up in the CLNM (+) and CLNM (-) groups are shown in **Figure 10**. The proportions of FT3< 3.1 pmol/L and FT4< 12 pmol/L reached its maximum at 3 days postoperatively and were higher in the CLNM (+) group than in the CLNM (-) group, then gradually decreased to 0. TSH > 4.2 uIU/ml reached highest value at 1 month postoperatively, with 33.49% and 28.42% in the CLNM (+) and CLNM (-) groups, respectively, and then gradually declined. The proportion with Tg< 3.5 ng/ml increases dramatically at 1 month postoperatively. TGAb and TPOAb varied steadily, with an overall gradual decline in the portion above the normal range.

**Figure 11:**
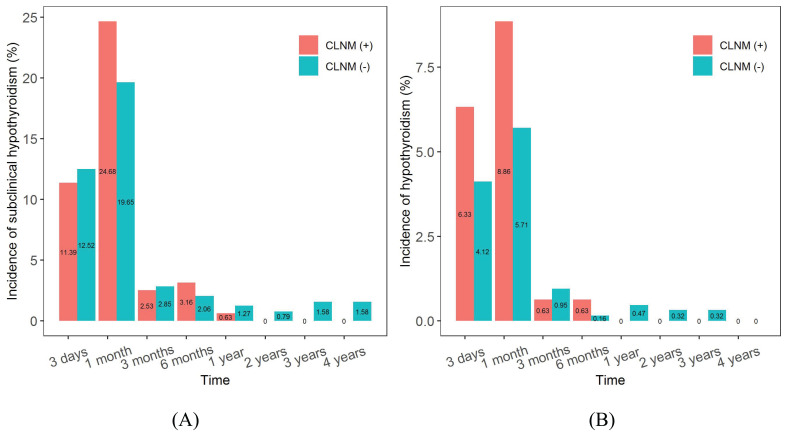
Incidences of subclinical hypothyroidism and hypothyroidism after surgery in the CLNM (+) and CLNM (-) groups of PTMC patients with 4-year follow-up. **(A)** subclinical hypothyroidism, **(B)** hypothyroidism.

The incidences of subclinical hypothyroidism and hypothyroidism tended to increase and then decrease in both the CLNM (+) and CLNM (-) groups, as shown in **Figures 11A, B**. Subclinical hypothyroidism occurred predominantly within 6 months postoperatively and reached a maximum at 1 month postoperatively, with a higher incidence in the CLNM (+) group than in the CLNM (-) group, at 24.68% and 19.65%, respectively. The overall incidence were 42.41% (67 of 158 patients) in the CLNM (+) group and 42.31% (267 of 631 patients) in the CLNM (-) group. Hypothyroidism appeared mainly within 3 months postoperatively and peaked at 1 month postoperatively, with a incidence of 8.86% in the CLNM (+) group higher than that of 5.71% in the CLNM (-) group. The overall incidence were 16.46% (26 of 158 patients) in the CLNM (+) group and 12.04% (76 of 631 patients) in the CLNM (-) group.

## Discussion

The incidence of thyroid cancer has risen rapidly in most parts of the world over the past 30 years, and more markedly in developed countries (20, 21). PTC is the most common type of differentiated thyroid malignancy and it represents about 90% of all thyroid cancers (22, 23). PTMC is a subtype of PTC, defined as PTC with tumors up to 10 mm in maximum diameter (5). The number of PTMC patients is increasing due to the use of high-resolution ultrasound (USG) and fine needle biopsy (24). Although in the majority of patients PTMC is usually associated with a good prognosis, PTMC with CLNM is not uncommon. In addition, several studies have demonstrated lymph node metastases at presentation, with locoregional recurrence during the follow-up period (25, 26). Some researchers recommend the use of prophylactic CLND in patients with PTMC (27, 28), but this approach remains a matter of debate. Therefore, determining the predictors of CLNM is very important to avoid unnecessary CLND in patients with PTMC. To improve the diagnostic accuracy of PTMC, many authors have used a combination of clinicopathological and USG features (29). High-resolution ultrasonography is helpful in evaluating LNM (30). However, despite the high diagnostic accuracy in detecting the LLNM, the accuracy of ultrasonography in preoperative detection of CLNM is limited (31). Consequently, preoperative assessment of CLNM in PTMC patients is challenging. Thus, a tool that helps to quantify the risk of nodal metastasis may facilitate preoperative decision-making (32). For these reasons, several authors have developed different predictive models to assess the risk of CLNM in patients with PTMC that combine clinical, analytical, and ultrasound variables (18, 32). These may help clinicians and patients improve clinical decision-making.

Nomograms are statistical tools that are ideally suited to individualizing risk assessment and they have been used for a variety of situations (33). Nomograms have been shown to outperform even recognized by experts in the field of oncology (34, 35). In this study, we included the clinical characteristics, biochemical profiles, ultrasonographic and cytologic features in a nomogram and predicted the risk of CLNM in patients with PTMC. The original data were randomly divided into a training group (model construction) and a validation group (model validation), the verification results showed that both have good risk prediction ability. The model was assessed to have good discrimination and calibration capabilities by plotting ROC curves and calibration curves. Meanwhile, DCA also confirmed the clinical utility value of the nomogram. This study suggested that age, gender, calcification, diameter, multifocality, ETE, ECLN, LLNM, location_UML, BMI, and CEA were independent risk factors for CLNM in patients with PTMC. Among them, male gender, 1 ≥ diameter ≥ 0.5cm, calcification, multifocality, ETE, LLNM, ECLN, location in lower pole and CEA correlated positively with CLNM. Age and BMI correlated negatively with CLNM. LLNM was the most strongly correlated indicator. The nomogram we constructed can well predict the risk of CLNM in patients with PTMC, which helps clinicians make decisions regarding the extent of surgical resection.

The AUC value is an important criterion for evaluating the performance of a model and is mainly used to demonstrate the discriminatory ability of the model. Yang et al. (36) established a nomogram to predict CLNM in PTMC patients with an AUC = 0.69, but there existed deficiencies in the study, for example, the small sample (291 PTMC patients) and the absence of validation. Wang et al. (37) estimated in a large retrospective study that the AUC of the nomogram predicting the risk of CLNM in patients with PTMC was 0.711. In our study, the AUC were 0.73, and 0.75 in the training and validation groups, respectively. Our estimated AUC values are significantly higher than the findings of some scholars, which fully demonstrates the good discriminatory ability of our constructed nomogram in detecting CLNM in patients with PTMC. It is worth mentioning that the AUC value of the constructed nomogram model in the validation group was 0.02 higher than that in the training group, which indicates that the model has better predictive performance in the validation group than in the training group, suggesting that the model is not overfitted, has some generalization ability, and can be better used in the dataset to predict the risk of CLNM in patients with PTMC. However, the results of this study also have the limitation that the AUC value did not exceed 0.80, which suggests that the model we constructed could further improve its prediction performance, such as by adding gene mutation analysis, and by combining with future biological assessments including liquid biopsies and other possible risk factors.

Among the independent risk factors in this CLNM prediction model, LLNM was the factor with the greatest risk weight (OR = 5.43). our findings is similar to those of Wang et al. (37). Therefore, when LLNM is detected during thyroid surgery, surgeons need to pay more attention to the central lymph nodes and evaluate the presence of CLNM. For patients with PTMC, tumor size was positively correlated with the risk of CLNM, the greater the diameter of the cancer lesion, the higher the risk and the worse the prognosis. However, the existing studies have no unified standard for the diagnostic cut-off value of cancer lesion size (37–39). Wang et al. (37) found that tumor size 0.5-1.0 cm was an independent predictor of central CLNM. Sun et al. (38) believed that PTMC patients with tumor diameter > 6 mm have higher rates of CLNM compared with patients with a tumor diameter ≤ 6 mm. Lim et al. (39) suggested that a nodule of 7 mm in size should be chosen as the diagnostic cut-off value. Our study also concluded that a diameter ≥ 0.5 cm is the threshold for CLNM, and logistic multivariate regression analysis produces meaningful results (OR = 1.78). Multifocality was previously found to be associated with LNM in the central or lateral compartment (40). Multifocality is an indicator of the aggressiveness of PTC tumors, showing a higher tendency for regional LNM (41). Consistently, in our results on PTMC, multifocality was a higher risk factor for CLNM (OR = 1.66). Therefore, multifocality may be associated with the state of disease progression, including risk stratification, management guidelines, and post-treatment monitoring in patients with PTMC. ETE has been recognized as an important prognostic factor in PTC (10, 35). ETE indicates poor prognosis for patients with PTC. However, the relationship between ETE and LNM has been examined in relatively small retrospective cohort studies (42, 43). As indicated in the study by Zhang et al. (44) and Sun et al. (38), ETE was a potential predictor for CLNM in patients with PTMC. Our findings approximated theirs (OR = 1.47). The above risk factors were primarily derived from pathologic testing of patients with PTMC.

Our study showed that ultrasound findings of ECLN, tumors located at the lower pole, and calcification were also high risk factors for developing CLNM. The findings of Mohamed et al. (45) showed that the presence of ECLN increased the predictive value of diagnosing PTC and suspicious thyroid nodules. The presence of an ECLN on the preoperative neck USG can provide valuable information to help surgeons determine the optimal surgical treatment for patients with suspected thyroid nodules (46). Our study also confirmed that ECLN is an independent risk factor for CLNM (OR = 1.66). We believe that the above studies will encourage further research investigating the association between ECLN and CLNM in patients with PTMC. Zhang et al. (47) hypothesized that the risk of LNM for PTC nodules at different locations might be associated with blood reflux. PTC cells in the upper region are more likely to transport to the lateral lymph nodes through the lymph flow along the superior thyroid artery (48). Hence, tumors located in the upper thyroid lobe confer a lower risk of developing CLNM, and conversely, tumors located in the lower thyroid lobe confer a high risk of developing CLNM (49, 50). Calcification is an important ultrasound feature of PTC (51). Microcalcifications are considered to be the most specific sonographic indicator in the diagnosis of PTC (52, 53). In this study, we found that calcification on the USG image is a risk factor for CLNM (OR = 1.34). Calcification is a calcium salt deposition caused by the proliferation of blood vessels and fibers, reflecting the rapid growth of cancer cells. Therefore, if calcifications are found in the nodules, the lymph node status in the central region should be assessed more carefully.

**Figure 12:**
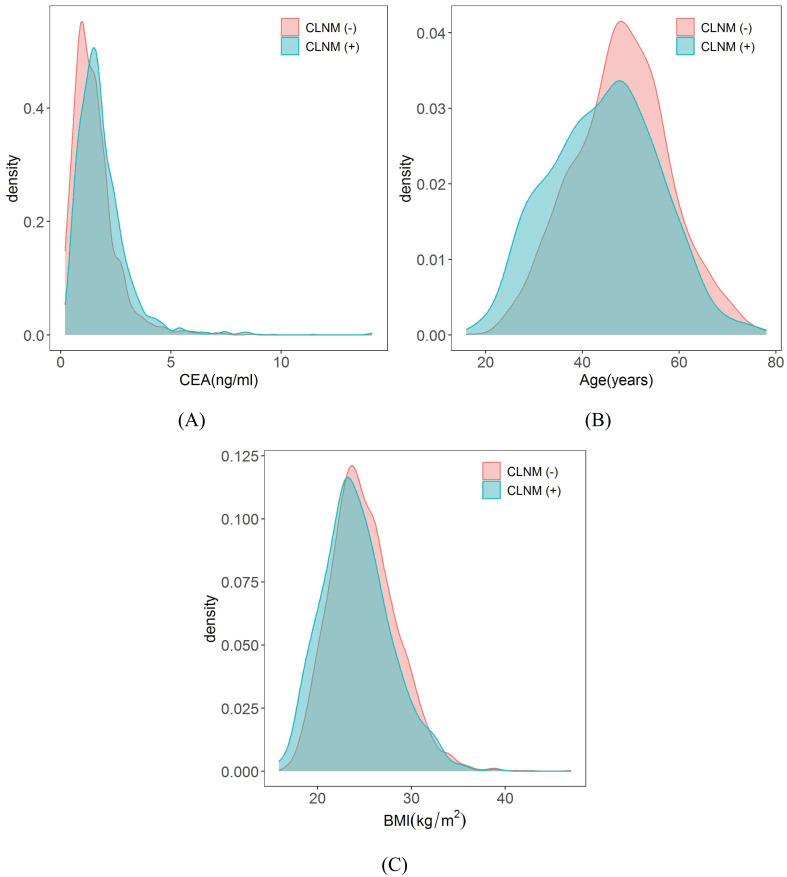
Probability density distribution of CEA, Age and BMI in CLNM (+) group and CLNM (-) group respectively. **(A)** CEA, **(B)** Age, and **(C)** BMI.

Among the biochemical variables and basic patient information, the male gender, CEA, young age and lower BMI are also independent risk factors of CLNM in patients with PTMC. It was confirmed in the study by Li et al. (54) that males had higher average annual percentage changes in thyroid cancer incidence and mortality rates than females. There are several explanations for these differences between males and females. Firstly, women undergo thyroid tests more frequently, and they are more likely to participate in medical treatment. Furthermore, this difference may be due to physiological differences between males and females (55, 56). In agreement with results of several previous studies (9, 57), male gender (OR = 1.60) was found to be an independent predictor for CLNM in PTMC. CEA is a broad spectrum tumor marker that has been found to be elevated in some patients with PTC (58). For example, Yan et al. (59) indicated that the combination of serum CEA, Tg, and CK 18 significantly improved the diagnostic efficiency of PTC patients. A retrospective cohort study of two clinical centers showed that CEA > 1 ng/mL was significantly associated with CLNM (18). Our research also confirmed that patients in CLNM (+) group had higher levels of CEA (1.66 ± 1.19 ng/ml) than those in the CLNM (–) group (1.53 ± 1.02 ng/ml) and the difference was statistically significant (*P* = 0.017), and the probability density distribution of CEA is shown in **Figure 12A**. Age is a well-established prognostic factor for thyroid cancer survival, and it is included in the American Joint Committee on Cancer (AJCC) thyroid cancer-staging system (59, 60). Ito et al. (61) reported that being young was an independent predictor of progression in PTMC under observation, including novel LNM. Our study also suggested that younger people were more likely to develop CLNM, and the age of the CLNM (+) group (44.57 ± 11.13 years) was lower than that of the CLNM (-) group (48.04 ± 10.06 years), with a statistically significant difference (*P<* 0.001), and the probability density distribution of the age is shown in **Figure 12B**. However, we did not stratify for age, which still needs to be investigated in studies with larger sample sizes. Our nomogram model quantitatively showed that patients with low BMI had a higher risk of developing CLNM, with a lower BMI in the CLNM(+) group (24.57 ± 3.61 kg/m^2^) than in the CLNM(-) group (24.94 ± 3.51 kg/m^2^), the difference was statistically significant (*P* = 0.009), and the probability density distribution of BMI are shown in **Figure 12C**. The results were consistent with Zhao et al. (62). Of course, some scholars have suggested that BMI is positively associated with CLNM (63). But the sample size of our study was larger than theirs. This finding may be due to the small sample size in multivariate analysis of potential comprehensive risk factors in real clinical scenarios, which may lead to bias in individual factors. To address this issue, we will continue to expand the sample size and conduct multi-center studies to further validate these results.

Currently, studies on the follow-up of thyroid function after thyroid cancer surgery have been reported, some of which explored the incidence, risk factors, and clinical features of hypothyroidism after thyroid cancer resection, but did not analyze in depth the differences in thyroid function between the CLNM (+) and CLNM (-) groups. In this study, we systematically analyzed trends in complete indicators of thyroid function in the CLNM (+) and CLNM (-) groups in 789 postoperative thyroid cancer patients followed for 4 years. Bae et al. (19) analyzed thyroid function in 369 postoperative thyroid cancer patients follow-up for 2 years and demonstrated that TSH concentration gradually increased over 3-6 months and then decreased to a level higher than preoperative level, whereas FT4 concentration showed opposite trend in consecutive measurements. In our study, the overall trend of TSH and FT4 levels were almost identical to their results. Postoperative changes in FT4 and TSH levels affect the incidence of postoperative complications subclinical hypothyroidism and hypothyroidism.

Hypothyroidism after thyroid cancer resection is a major clinical problem, as a significant number of patients require thyroid hormone replacement therapy. Verloop et al. (64) reported through a systematic review that the overall risk of hypothyroidism after lobectomy was 22% (range 7-49%). Studies by Ahn et al. (65) and Park et al. (66) illustrated that the incidence of subclinical hypothyroidism after hemithyroidectomy was as high as 55.8% and 64.2%, respectively. Subclinical hypothyroidism developed in the early postoperative period (1-3 months postoperatively) in most cases (84.5%). Our research showed that the incidence of subclinical hypothyroidism and hypothyroidism was significantly higher in the CLNM (+) group than in the CLNM (–) group at 1 month postoperatively, after which the incidence in the CLNM (+) group decreased dramatically to zero. The overall incidence of hypothyroidism was 18.35% in the CLNM (+) group and 13.79% in the CLNM (-) group. The results suggested that our findings are within the range of Verloop et al. (64), indicating that they are reasonable. We analyzed the differences in thyroid function between the CLNM (+) and CLNM (-) groups, which were mainly attributed to the extent of surgical removal of the thyroid gland, comparative results after total thyroidectomy and hemithyroidectomy are presented in the **Supplementary Table S1**, **Supplementary Figures S1, S2**. The proportions of patients who underwent total thyroidectomy in the CLNM (+) and CLNM (-) groups were 63.92% (101 out of 158 patients) and 43.58% (275 out of 631 patients), respectively, which implied that patients in the CLNM (+) group were more prone to thyroid dysfunction in the postoperative period, and required levothyroxine supplementation to regulate the thyroid hormone levels.

Postoperative complications of PTMC also include laryngeal nerve palsy, hypoparathyroidism, blood loss, hematoma, and wound infection, which were not comprehensively analyzed in this study and were only associated with subclinical hypothyroidism and hypothyroidism that can be calculated by follow-up thyroid function indicators. However, Ultrasonic scalpel and the Ligasure have been proven to be safe, effective, useful, and time-saving as alternatives to the traditional surgical thyroid suture ligation techniques. They simplify total thyroidectomy, eliminating the need for clamp-and-tie maneuvers and achieving efficient hemostasis (67, 68). This means a shorter operation time and a lower postoperative complication rate. Relevant studies will be further analyzed in a comprehensive and systematic manner.

In this study, the nomogram model based on clinical, biochemical, ultrasound and pathological features had good predictive performance. Meanwhile, the calibration plots and DCA results also proved that the prediction model has good discriminatory ability and clinical application value. The present study still has the following deficiencies: (1) The internal validation of the nomograms in this study came from the same hospital and lacked external validation, which may have resulted in case selection bias. Therefore, further analysis and evaluation should be carried out in combination with multicenter large-sample clinical data. (2) A larger sample size of clinical data is still needed to improve the validity and reliability of the model. (3) Some data in this study need further stratification and quantitative research. (4) Lastly, our study is retrospective, and prospective studies in larger patient populations are required to identify and validate this risk prediction model to improve clinical management.

## Conclusion

Accurate preoperative identification of CLNM is essential for surgical protocol establishment for patients with PTMC. Therefore, it is necessary to search for risk factors for CLNM in PTMC patients. In this study, we proposed a nomogram model combining clinical, biochemical, ultrasound and pathological features. With the visualization of a nomogram model, it may help clinical practitioners inform screening and early diagnosis of CLNM by Incorporating patient information into risk prediction models and calculating risk scores to assess the risk of CLNM in patients with PTMC. Regular follow-up of postoperative thyroid function indicators and the incidence of complications in PTMC patients can provide clinicians with a reference for developing postoperative treatment plans.

## Data Availability

The raw data supporting the conclusions of this article will be made available by the authors, without undue reservation.

## Glossary

PTC: papillary thyroid carcinoma
PTMC: papillary thyroid microcarcinoma
CLNM: central lymph node metastasis
LASSO: least absolute shrinkage and selection operator
BMI: body mass index
USG: ultrasonography
IH: intranodular hyperechoic
ETE: extrathyroidal extension
ECLN: enlarged central lymph nodes
ATA: American Thyroid Association
CLND: central lymph node dissection
TG: triglyceride
CHOL: cholesterol
FT3: free triiodothyronine
FT4: free thyroxine
TSH: thyroid stimulating hormone
TRAb: thyrotropin receptor antibody
TGAb: anti-thyroglobulin antibodies
TPOAb: thyroid peroxidase antibodies
Tg: thyroglobulin
AFP: alpha fetoprotein
CEA: carcinoembryonic antigen
Ct: calcitonin
CDFI: color doppler flow imaging
AJCC: American Joint Committee on Cancer
ROC: receiver operating characteristic
AUC: area under the curve
DCA: decision curve analysis
ELR: extreme low risk
LR: low risk
MR: moderate risk
HR: high risk
CI: confidence interval
OR: odds ratio
