# Supplementary material for: Risk nomogram for papillary thyroid microcarcinoma with central lymph node metastasis and postoperative thyroid function follow-up
Source: Front Endocrinol (Lausanne). 2024 Oct 28;15:1395900. doi: 10.3389/fendo.2024.1395900 (PMC11550994; doi:10.3389/fendo.2024.1395900)
Supplement: Supplementary file 1 [file DataSheet1.docx]

**Supplementary Materials**

**Follow-up results of postoperative FT4 and TSH levels**

We compared FT4 and TSH levels in total thyroidectomy patients and hemithyroidectomy patients 4 years after surgery, respectively, as shown in **Table S1** and **Figure S1A, B**. FT4 levels gradually increased and reached a relatively stable state 3 months after surgery. There were significant differences in FT4 levels between the two groups within 3 years postoperatively (*P* < 0.05), and FT4 levels were higher in patients with total thyroidectomy than in patients with hemithyroidectomy after 3 months postoperatively. TSH levels increased rapidly in total thyroidectomy patients for 1 month after surgery and then decreased abruptly. It gradually decreased in hemithyroidectomized patients. TSH levels were significantly higher in total thyroidectomy patients than in hemithyroidectomy patients within 3 months after surgery, and the difference was statistically significant (*P* < 0.05).

**Table S1** FT4 and TSH levels at follow-up within 4 years after thyroidectomy.

| **Variables** | **Time** | **Total**  **n = 789** | **Total thyroidectomy**  **n = 376** | **Hemithyroidectomy**  **n = 413** | ***t*** | ***P*** |
| --- | --- | --- | --- | --- | --- | --- |
| FT4 |  |  |  |  |  |  |
|  | Preoperative | 15.90±2.52 | 15.98±2.36 | 15.83±2.66 | -0.942 | 0.346 |
|  | 3 days | 16.21±3.95 | 15.86±3.98 | 16.52±3.90 | 2.640 | 0.008 |
|  | 1 month | 17.47±4.27 | 16.99±4.65 | 17.92±3.85 | 3.373 | 0.001 |
|  | 3 months | 20.09±4.44 | 20.56±4.88 | 19.66±3.96 | -2.789 | 0.005 |
|  | 6 months | 20.50±4.46 | 21.42±4.51 | 19.67±4.24 | -5.216 | <0.001 |
|  | 1 year | 20.13±4.07 | 20.98±4.18 | 19.31±3.78 | -5.260 | <0.001 |
|  | 2 years | 19.75±3.70 | 20.63±4.00 | 18.84±3.11 | -5.436 | <0.001 |
|  | 3 years | 19.47±3.78 | 20.13±4.12 | 18.74±3.23 | -3.371 | 0.001 |
|  | 4 years | 19.71±3.67 | 20.18±4.21 | 19.19±2.89 | -1.220 | 0.226 |
| TSH |  |  |  |  |  |  |
|  | Preoperative | 2.63±1.60 | 2.65±1.71 | 2.50±1.50 | -1.402 | 0.161 |
|  | 3 days | 2.68±3.20 | 3.00±3.82 | 2.40±2.48 | -2.892 | 0.004 |
|  | 1 month | 6.84±13.19 | 11.84±17.26 | 2.27±4.18 | -11.636 | <0.001 |
|  | 3 months | 2.36±8.52 | 3.53±11.66 | 1.30±3.55 | -3.554 | <0.001 |
|  | 6 months | 1.65±7.13 | 2.07±9.42 | 1.28±4.02 | -1.403 | 0.161 |
|  | 1 year | 1.49±6.96 | 2.10±9.83 | 0.98±1.13 | -1.891 | 0.068 |
|  | 2 years | 1.01±2.68 | 0.80±1.61 | 1.22±3.45 | 1.694 | 0.091 |
|  | 3 years | 1.44±3.42 | 1.58±3.93 | 1.27±2.75 | -0.833 | 0.405 |
|  | 4 years | 1.41±3.46 | 1.71±4.48 | 1.07±1.69 | -0.835 | 0.407 |


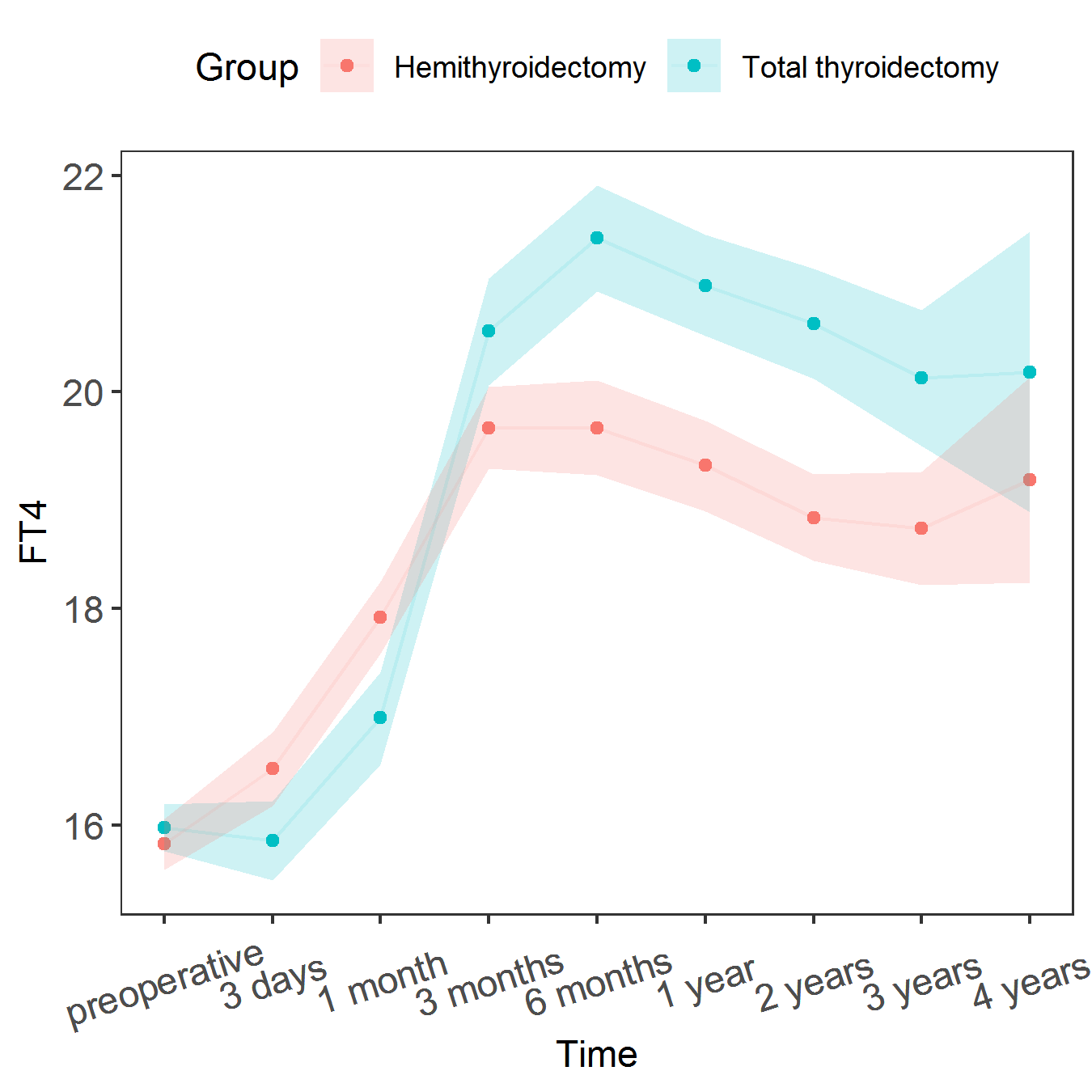

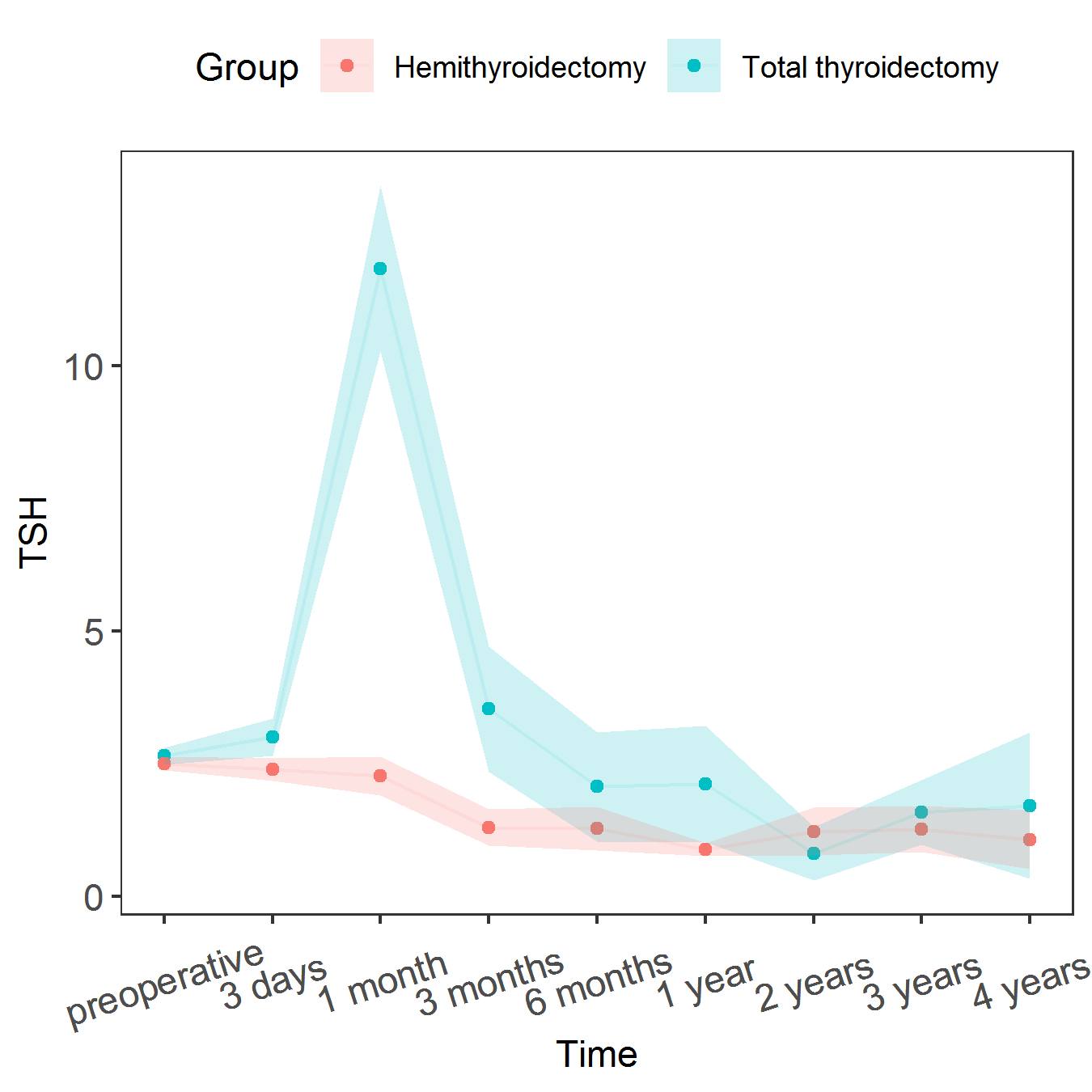


(A) (B)

**FIGURE S1** Trends in FT4 and TSH levels at follow-up within 4 years after thyroidectomy.

**Incidence of postoperative complications**

The incidence of both subclinical hypothyroidism and hypothyroidism increased to a maximum at 1 month postoperatively in patients with total thyroidectomy and then decreased. It gradually decreased in hemithyroidectomized patients. The overall incidence of subclinical hypothyroidism was 42.33% (334 out of 789 patients), with 60.11% (226 out of 376 patients) in patients with total thyroidectomy and 26.15% (108 out of 413 patients) in patients with hemithyroidectomy (**Figure S2A**). The overall incidence of hypothyroidism was 12.93% (102 out of 789 patients), of which the incidence in patients with total thyroidectomy and hemithyroidectomy were 22.34% (84 out of 376 patients) and 4.36% (18 out of 413 patients), respectively (**Figure S2B**). This indicated that the incidence of both subclinical hypothyroidism and hypothyroidism were higher in total thyroidectomy patients than in hemithyroidectomy patients.


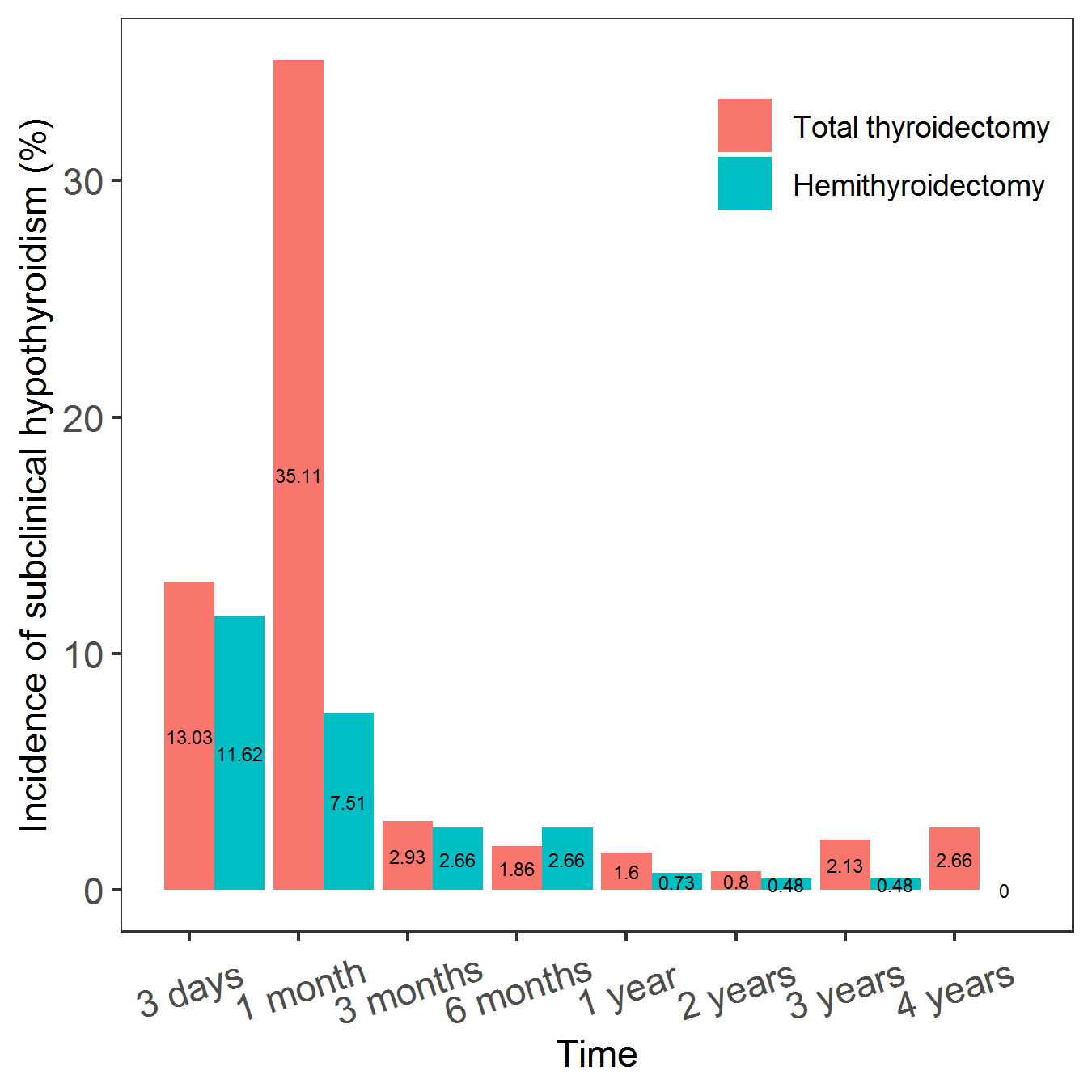

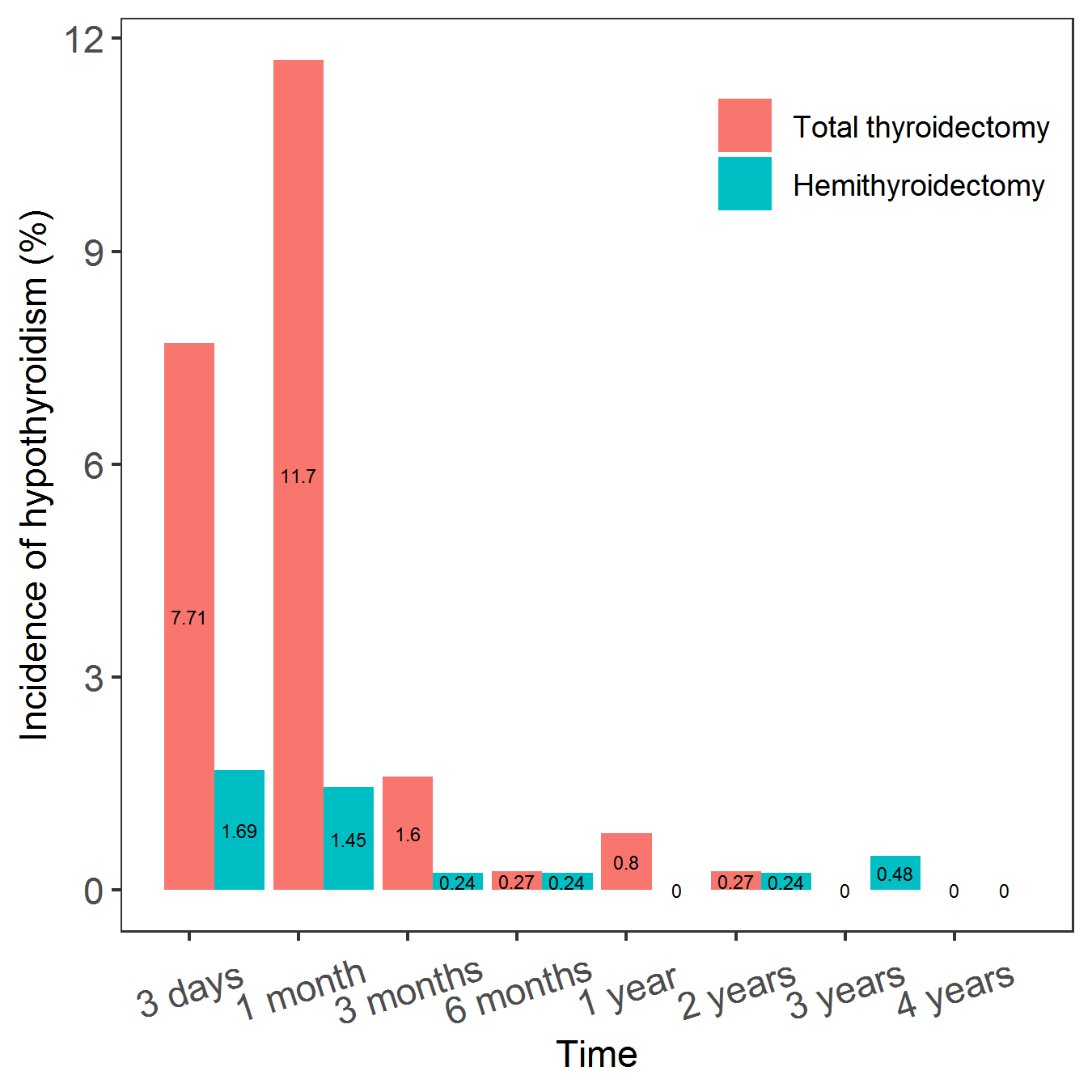


(A) (B)

**FIGURE S2** Incidence of subclinical hypothyroidism and hypothyroidism 4 years after surgery.
